# Exosomal miR-125b-5p deriving from mesenchymal stem cells promotes tubular repair by suppression of p53 in ischemic acute kidney injury

**DOI:** 10.7150/thno.54550

**Published:** 2021-03-11

**Authors:** Jing-Yuan Cao, Bin Wang, Tao-Tao Tang, Yi Wen, Zuo-Lin Li, Song-Tao Feng, Min Wu, Dan Liu, Di Yin, Kun-Ling Ma, Ri-Ning Tang, Qiu-Li Wu, Hui-Yao Lan, Lin-Li Lv, Bi-Cheng Liu

**Affiliations:** 1 Institute of Nephrology, Zhong Da Hospital, Southeast University School of Medicine, Nanjing 210009, China.; 2 Department of Medicine and Therapeutics, Li Ka Shing Institute of Health Sciences, Liu Che Woo Institute of Innovative Medicine, Chinese University of Hong Kong, Hong Kong SAR 999077, China.

**Keywords:** mesenchymal stem cell, exosomes, miR-125b-5p, tubular repair, acute kidney injury

## Abstract

Mesenchymal stem cells-derived exosomes (MSC-exos) have attracted great interest as a cell-free therapy for acute kidney injury (AKI). However, the *in vivo* biodistribution of MSC-exos in ischemic AKI has not been established. The potential of MSC-exos in promoting tubular repair and the underlying mechanisms remain largely unknown.

**Methods:** Transmission electron microscopy, nanoparticle tracking analysis, and western blotting were used to characterize the properties of human umbilical cord mesenchymal stem cells (hucMSCs) derived exosomes. The biodistribution of MSC-exos in murine ischemia/reperfusion (I/R) induced AKI was imaged by the IVIS spectrum imaging system. The therapeutic efficacy of MSC-exos was investigated in renal I/R injury. The cell cycle arrest, proliferation and apoptosis of tubular epithelial cells (TECs) were evaluated *in vivo* and in HK-2 cells. The exosomal miRNAs of MSC-exos were profiled by high-throughput miRNA sequencing. One of the most enriched miRNA in MSC-exos was knockdown by transfecting miRNA inhibitor to hucMSCs. Then we investigated whether this candidate miRNA was involved in MSC-exos-mediated tubular repair.

**Results:** *Ex vivo* imaging showed that MSC-exos was efficiently homing to the ischemic kidney and predominantly accumulated in proximal tubules by virtue of the VLA-4 and LFA-1 on MSC-exos surface. MSC-exos alleviated murine ischemic AKI and decreased the renal tubules injury in a dose-dependent manner. Furthermore, MSC-exos significantly attenuated the cell cycle arrest and apoptosis of TECs both *in vivo* and *in vitro*. Mechanistically, miR-125b-5p, which was highly enriched in MSC-exos, repressed the protein expression of p53 in TECs, leading to not only the up-regulation of CDK1 and Cyclin B1 to rescue G2/M arrest, but also the modulation of Bcl-2 and Bax to inhibit TEC apoptosis. Finally, inhibiting miR-125b-5p could mitigate the protective effects of MSC-exos in I/R mice.

**Conclusion:** MSC-exos exhibit preferential tropism to injured kidney and localize to proximal tubules in ischemic AKI. We demonstrate that MSC-exos ameliorate ischemic AKI and promote tubular repair by targeting the cell cycle arrest and apoptosis of TECs through miR-125b-5p/p53 pathway. This study provides a novel insight into the role of MSC-exos in renal tubule repair and highlights the potential of MSC-exos as a promising therapeutic strategy for AKI.

## Introduction

Acute kidney injury (AKI) is a common syndrome defined by abrupt renal function decline. AKI occurs in approximately 10%-15% hospitalized patients and in even more than 50% intensive care unit patients 1,2. More importantly, AKI is an independent predictor of in-hospital mortality and has a high risk of progression to chronic kidney disease or end-stage renal disease for those survivors 1. Finding specific therapeutic strategies for established AKI is desperately needed 3. Renal ischemia/reperfusion (I/R) injury is a main cause of AKI, characterized by lethal and sublethal injuries of renal tubules 4. Researches have demonstrated that maladaptive and incomplete repair of renal tubules led to the progress of renal fibrosis 5, 6. Thus, protecting renal tubular epithelial cells (TECs) from ischemic insult and promoting tubular repair are critical for improving the outcomes of ischemic AKI 4, 7.

Mesenchymal stem cells (MSCs), characterized by the abilities of self-renewal, differentiation, immunomodulation, and trophic support, are essential in regenerative medicine owing to the capacity to create a microenvironment conducive to the repair of injured tissues 8. Recently, a Phase 1a escalating clinical trial showed that autologous MSCs could not only increase blood flow and glomerular filtration rate, but also attenuate inflammation in post-stenotic kidneys 9. However, the short and long-term safety concerns of MSC-based therapy, for example, the risk of embolization, immunogenicity, and tumor formation, are the main obstacles for its clinical applications 10-12.

Exosomes, a major class of extracellular vesicles (EVs) secreted by almost all kinds of cells, mediate intercellular communications by transferring bioactive molecules, such as mRNAs, non-coding RNAs, lipids, and proteins 13,14. It is worth noting that EVs derived from MSCs have been proposed as an alternative to MSC-based therapy for several diseases 15. In the previous study, we found that exosomes derived from MSCs (MSC-exos) could significantly attenuate cisplatin-induced murine AKI through inhibiting inflammation 16. Although the exact mechanisms are largely assumptive, anti-inflammation, anti-apoptosis, immunomodulation, and promoting proliferation are all conducive to the treatment of AKI 17. Exploring the underlying mechanism of MSC-exos in the treatment of AKI, especially for tubular repair, is crucial to the clinical application of this novel therapy.

Numerous studies have proved that the enriched specific miRNAs of MSC-exos might account for the pro-repair effects in AKI and other diseases 3, 18. Wu et al. 19 suggested that miR-100-5p-abundant MSC-exos attenuated cartilage injury via inhibition of mammalian target of rapamycin in osteoarthritis. Duan et al. 20 found that adipose mesenchymal stem cell-derived EVs containing miR-26a-5p protected against diabetic nephropathy. In addition, depletion of miRNAs from EVs significantly weakened their regenerative potential in glycerol-induced AKI, suggesting the vital role of miRNA in the therapeutic effect of MSC-EVs 21. Consequently, the objective of this study was to examine the efficacy of MSC-exos in tubular repair and the role of specific miRNAs during this process.

In this study, we demonstrated the biodistribution and therapeutic benefits of MSC-exos in murine ischemic AKI. MSC-exos were accumulated in the injured kidney, especially in proximal tubules, which was credited to the integrins on the surface of exosomes. Treatment with MSC-exos markedly ameliorated renal I/R injury through limiting the cell cycle arrest and apoptosis of TECs. Specifically, after profiling the exosomal miRNAs of MSC-exos by high-throughput miRNA sequencing (miRNA-seq), we demonstrated that MSC-exos exerted renal protective effect at least partially through miR-125b-5p/p53 signaling mediated tubular repair. Our findings strongly supported MSC-exos was an innovative therapeutic approach for tubular repair after AKI.

## Methods

### Isolation and purification of MSC-exos

Human umbilical cord mesenchymal stem cells (hucMSCs, donated by Wingor Biotechnology Co, Ltd) were cultured with media mixed with mesenchymal stem cell medium (ScienCell) and DMEM/F12 (Gibco), supplemented with 5% fetal bovine serum (FBS; ScienCell). The identification of hucMSCs was determined as previously described 16. To prepare MSC-exos, hucMSCs (Passage 2-6) were cultured with FBS-free media for 48 h, and the supernatants were subsequently collected for MSC-exos extraction. MSC-exos were isolated by gradient centrifugation and filtration 16. In brief, the supernatants were centrifuged at 2000×g for 20 min, 13,500×g for 30 min, filtrated with a 0.22-μm filter and ultracentrifuged at 200,000×g for 120 min (Beckman Coulter Optima L-80 XP) at 4 °C. The exosome pellets were resuspended in sterile phosphate buffer saline (PBS) and filtered using a 0.22-μm filter once again. Purified MSC-exos were resuspended in PBS and stored at -80 °C.

### Characterization of MSC-exos

The morphology of MSC-exos was observed by transmission electron microscopy (TEM; HITACHI). The particle size and zeta potential 22 were detected by Nanoparticle tracking analysis (NTA; ZetaView PMX 110). Surface markers including exosomal markers (Alix, CD63, Tsg101, CD9), Golgi marker (GM130), and MSC-associated protein (CD44), were analyzed by Western blotting.

### Labeling of MSC-exos

To obtain labeled MSC-exos, the donor hucMSCs were incubated with 1,1'-dioctadecyl-3,3,3',3'-tetramethylindodicarbocyanine perchlorate (DiD; Invitrogen) or 1,1′-dioctadecyl-3,3,3′,3′-tetramethylindocarbocyanine perchlorate (DiI; Invitrogen) and washed twice with sterile PBS to remove excess dye. After being cultured in FBS-free media for 48 h, the supernatants were used to isolate DiD-labeled MSC-exos or DiI-labeled MSC-exos in the same procedure as above 23. In this way, the free dye could be removed to the utmost extent. For blockade of integrins, DiD-labeled MSC-exos or DiI-labeled MSC-exos were incubated with anti-LFA-1 antibodies (20 μg/mL, BE0005, BioXCell) and anti-VLA-4 antibodies (20 μg/mL, BE0071, BioXCell) or rat IgG overnight at 4 °C 24.

### Animal models and therapeutic experiments

Male C57BL/6 mice (Vital River Laboratory Animal Technology Co., Ltd.,) were used at 8-10 weeks old with 21-24 g body weight. Experimental procedures were approved by the ethics committees for animal experimentation of Southeast University (No. 20190410016). Bilateral I/R injury was induced in mice by renal pedicle clamping for 30 min, as previously described 25. The body temperature of mice was controlled at 36.5-37 °C by a sensitive rectal probe. The sham mice were administered with exposure of kidneys but without clamping the renal pedicle. At 0 and 24 h after reperfusion, mice were administered intravenously with PBS, MSC-exos (50 μg), or MSC-exos (100 μg). Mice were euthanized at 48 h post-I/R injury. To investigate whether miR-125b-5p was involved in MSC-exos-mediated tubular repair *in vivo*, hucMSCs were transfected with miR-125b-5p inhibitors or negative control (NC) inhibitors. Then exosomes were isolated from the conditioned media. The exosomes isolated from miR-125b-5p inhibitor transfected MSC (miR-125b-5p^IN^-exos, 100 μg), NC-MSC (NC-exos, 100 μg) or PBS were administered intravenously at two time points after I/R insult: 0 h and 24 h (n = 5, respectively).

### *In vivo* biodistribution of MSC-exos

To assess tissue distribution of MSC-exos* in vivo*, DiD-labeled MSC-exos (100 μg) were injected intravenously into sham or I/R mice. Organs including heart, lung, liver, spleen, and kidneys were excised 12 h after injection and imaged by the IVIS spectrum imaging system (PerkinElmer) with the same conditions 26. In addition, the sectioned tissue of kidneys was stained with 4',6-diamidino-2-phenylindole (DAPI), and images were captured using a confocal microscope (Olympus). The number of nuclei surrounded with DiD-labeled MSC-exos was divided by the total amount of nuclei to quantify the distribution of MSC-exos. Five random tissue sections were selected per kidney.

### Cell culture

Both human renal tubular epithelial cell line HK-2 (American Type Culture Collection) and mouse tubular epithelial cells (mTECs; a gift from J. B. Kopp, National Institutes of Health) were cultured in DMEM/F12 supplemented with 1% penicillin-streptomycin and 10% FBS.

### Establishment of *in vitro* hypoxia model and treatment

Cells were plated in six-well plates and cultured until reached approximately 80-90% confluence for downstream experiments. Cells in hypoxia/reoxygenation (H/R) group were firstly incubated under hypoxic conditions (1% O_2_, 94% N_2_, and 5% CO_2_) for 12 h in glucose- and FBS-free culture medium, then transferred back to complete culture medium with oxygen for reoxygenation. In MSC-exos treatment group, cells were incubated with MSC-exos (10 μg or 20 μg) for 12 h during hypoxia.

### Cellular uptake of MSC-exos *in vitro*

DiI-labeled MSC-exos were respectively incubated with HK-2 cells, with or without H/R intervention for 6 h. To optimize and validate the MSC-exos uptake assay, HK-2 cells were incubated with DiI-labeled MSC-exos at 4 °C or 37 °C 27. HK-2 cells were washed with sterile PBS to remove uninternalized MSC-exos for three times and were performed by confocal microscope or flow cytometry (ACEA NovoCyte). The number of DiI-labeled MSC-exos (red dots) was counted in 20 random fields from each well. The total numbers averaged for each cell were used to evaluate its uptake of MSC-exos.

### Cell counting kit-8 (CCK-8) assay

The cell viability of HK-2 and mTECs was determined by CCK-8 assay (APExBIO) following the instructions. The absorbance at 450-nm wavelength was measured by a microplate reader (Thermo Fisher).

### Apoptosis analysis

The apoptosis in mice kidney sections was examined with terminal deoxynucleotidyl transferase-mediated dUTP nick end labeling (TUNEL) assay (Beyotime). The apoptosis of HK-2 cells was detected through annexin-V/propidium iodide (PI) staining (BD Biosciences) and TUNEL staining.

### Histology and immunofluorescence staining

Renal tissues were used for periodic acid-Schiff (PAS) staining. The tubular injury was semiquantitatively scored as follows 28: 0, no damage; 1, <25%; 2, 25~50%; 3, 50~75%; 4, >75%. The tubular injury score was calculated as the average score from 10 random sections. For immunohistochemistry staining, 4-μm-thick renal tissue sections were performed with primary antibodies against CD68 (ab955, Abcam), or CD3 (ab16669, Abcam) and analyzed using streptavidin peroxidase detection system (Maixin) as instructing by the protocols. For immunofluorescence analysis, tissue sections were incubated with primary antibodies against kidney injury molecular-1 (KIM-1; MA5-28211, Invitrogen), Ki-67(ab15580, Abcam), and p-H3 (ab14955, Abcam), followed by incubation with secondary antibodies (ab150114 and ab150077, Abcam). The proximal tubules were labeled by Lotus tetragonolobus lectin (LTL, FL-1321, vector labs) 29,30. The number of positive tubules and cells was counted under a confocal microscope in ten random fields per mouse in a blinded manner.

### Single-cell suspension of renal tubule segments isolation

Kidneys were harvested from control, I/R, or MSC-exos-treated mice, and the TECs were isolated by differential sieving as previously described 23, 31. Briefly, kidneys were removed and put into cold DMEM/F12 medium. The renal cortex was cut into 1-mm^3^-thick fragments and ground with an 80-mesh steel sieve, followed by filtration through 150-mesh steel sieves. The tubule segments under 150-mesh steel sieves were collected and digested by 3 mL DMEM/F12 supplemented with 1 mg/mL type IV collagenase (Worthington), 10 μg/mL DNase (Sigma-Aldrich), and 3% FBS (Gibco) for 30 min at 37 °C. Then, the cell suspension was filtered through 70 μm cell strainers (BD Biosciences) and washed with PBS containing 2 mM EDTA and 2% FBS. After removing red blood cells, the cell suspension was filtered through 40μm cell strainers (BD Biosciences). Finally, TECs were fixed in 70% ethanol (4 °C).

### Flow cytometry analysis of cell cycle

The HK-2 and mTECs were exposed to FBS-free medium (12 h) for synchronization. MSC-exos (20 μg) or equal volume of PBS were incubated with H/R treated cells. The cells were fixed in 70% ethanol (4 °C) overnight. Then, the fixed cells and mice TECs were washed twice with PBS and incubated in 1 mg/mL DNase-free RNase A (Sigma-Aldrich) and 50 μg/mL PI for 30 min. The distribution of the cell cycle (G0/G1, S, and G2/M) was detected using a flow cytometer.

### Cell transfection with miRNA inhibitor, small interfering RNA (siRNA) and plasmid

HucMSCs were transfected with miR-125b-5p inhibitor or negative control (NC) inhibitor (GenePharma) at a concentration of 100 nM using Lipofectamine 3000 (Invitrogen). The sequences of miR-125b-5p and NC inhibitor were as follows: miR-125b-5p inhibitor (5′-UCACAAGUUAGGGUCUCAGGGA-3′); NC inhibitor (5′-CAGUACUUUUGUGUAGUACAA-3′). HK-2 cells were transfected with siICAM-1 and siVCAM-1 (GenePharma) at a concentration of 80 nM using Lipofectamine 3000 (Invitrogen). The sequences of siICAM-1 and siVCAM-1 were as follows: (1) siICAM-1: sense: GCCUCAGCACGUACCUCUATT; anti-sense: UAGAGGUACGUGCUGAGGCTT. (2) siVCAM-1: sense: AAUGCAACUCUCACCUUAATT; anti-sense: UUAAGGUGAGAGUUGCAUUTT. A plasmid containing P53 expression gene (pEX-P53) was constructed on a pEX-1 backbone (GenePharma). HK-2 cells were transfected with 1500 ng plasmid DNA using Lipofectamine 3000 (Invitrogen) in accordance with the manufacturer's instructions.

### Detection of mRNA and miRNA

The total RNA from the kidney tissue, HK-2, hucMSCs, or exosomes was extracted with RNAiso (Takara). mRNA was quantified using PrimeScript reverse transcription reagent and real-time PCR (RT-PCR) kit with SYBR Green (Takara). Mature miRNAs were quantified with the real-time PCR detection kit using miRNA-specific primers (GeneCopoeia). All the primer sequences were listed in Table S1.

### BrdU proliferation assay

With different interventions, HK2 cells were incubated in medium containing 10 μM BrdU (ab142567, Abcam). Then, cells were fixed and treated with 0.3% Triton X-100 for 10 min. Primary antibody against BrdU (ab6326, Abcam) and secondary antibodies (ab150077, Abcam) were used. Cell nuclei were counterstained with DAPI for 5 min and visualized using a confocal microscope. The proportion of the cells incorporating BrdU was counted in 20 randomly selected fields per confocal dish.

### Fluorescence *in situ* hybridization (FISH)

Cy3-labeled probe sequences of miR-125b-5p were devised by Genepharma (Shanghai, China). 7-mm frozen kidney tissue sections were digested with protease K (20 μg/mL) and prehybridized in pre-hybridization buffer for 30 min (37 °C), followed by hybridization using Cy3-labeled miR-125b-5p probes overnight (37 °C). The sections were then washed with saline-sodium citrate buffer at 42 °C to remove the unhybridized probe. The image of FISH was captured under confocal microscope.

### miRNA library construction and sequencing

The library construction and sequencing of miRNA were conducted by OE Biotech Co., Ltd. miRNAs of MSC-exos were extracted by exoEasy Maxi Kit (QIAGEN) and quantified by Qubit 2.0 (Life Technologies). The integrity was confirmed by Agilent 2100 TapeStation (Agilent Technologies). A total of 50 ng exosomal RNA from each sample was used for small RNA library construction. Small RNA sequencing was conducted by HiSeq 2500 (Illumina) with reads lengths from 15 to 41 bp.

### Luciferase reporter assay

HK-2 cells were co-transfected with 3′UTR luciferase reporter constructs (3′UTR-NC, 3′UTR-TP53, 3′UTR-TP53-mutant), miRNA (miRNA-NC or miR-125b-5p), and Renilla luciferase using GP-transfect-Mate (GenePharma). After 48 h of transfection, the luciferase activity of cells was measured by a Dual Luciferase Assay Kit (Promega) and microplate reader (Tecan M1000).

### Western blotting

MSC-exos, cells and kidney tissues were lysed in RIPA lysis buffer (ThermoFisher) and the protein concentration was detected using BCA assay (ThermoFisher). Protein samples were subjected to Bis-Tris Gel (Invitrogen) and transferred onto PVDF membranes (Millipore). Membranes were blocked in NcmBlot blocking buffer (NCM Biotech) for 10 min and incubated overnight with primary antibodies as follows: anti-CD9 (ab92726, Abcam), anti-CD63 (sc-5275, Santa Cruz), anti-Tsg101 (sc-7964, Santa Cruz) anti-Alix (sc-53540, Santa Cruz), anti-GM130 (12480, Cell Signaling Technology), anti-CD44 (ab189524, Abcam), anti-ICAM-1 (MA5407, Invitrogen), anti-VCAM-1 (39036, Cell Signaling Technology), anti-LFA-1 (ab13219, Abcam), anti-integrin α4 (8440, Cell Signaling Technology), anti-integrin β1 (sc-374429, Santa Cruz), anti-PCNA (sc-56, Santa Cruz), anti-CDK1 (sc-54, Santa Cruz), anti-caspase-3 (ab13847, Abcam), anti-Cyclin B1(4138, Cell Signaling Technology), anti-p53 (2524, Cell Signaling Technology), anti-Bcl-2 (sc-7382, Santa Cruz), and anti-Bax (sc-20067, Santa Cruz). Secondary antibodies were used for detection by an ECL advanced system (GE Healthcare). Intensity values expressed as the relative protein expression were normalized to β-actin (AB2001, Abways) or GAPDH (AB2000, Abways).

### Statistical analysis

Data were expressed as mean ± standard deviation (SD). A two-tailed unpaired Student's *t*-test was performed for comparison between two groups. One-way ANOVA was used to compare among three or more groups, followed by Bonferroni correction for multiple comparisons. All analyses were conducted with SPSS 22.0. *P* < 0.05 was considered a significant difference.

## Results

### MSC-exos homing to injured kidney

**Figure 1:**
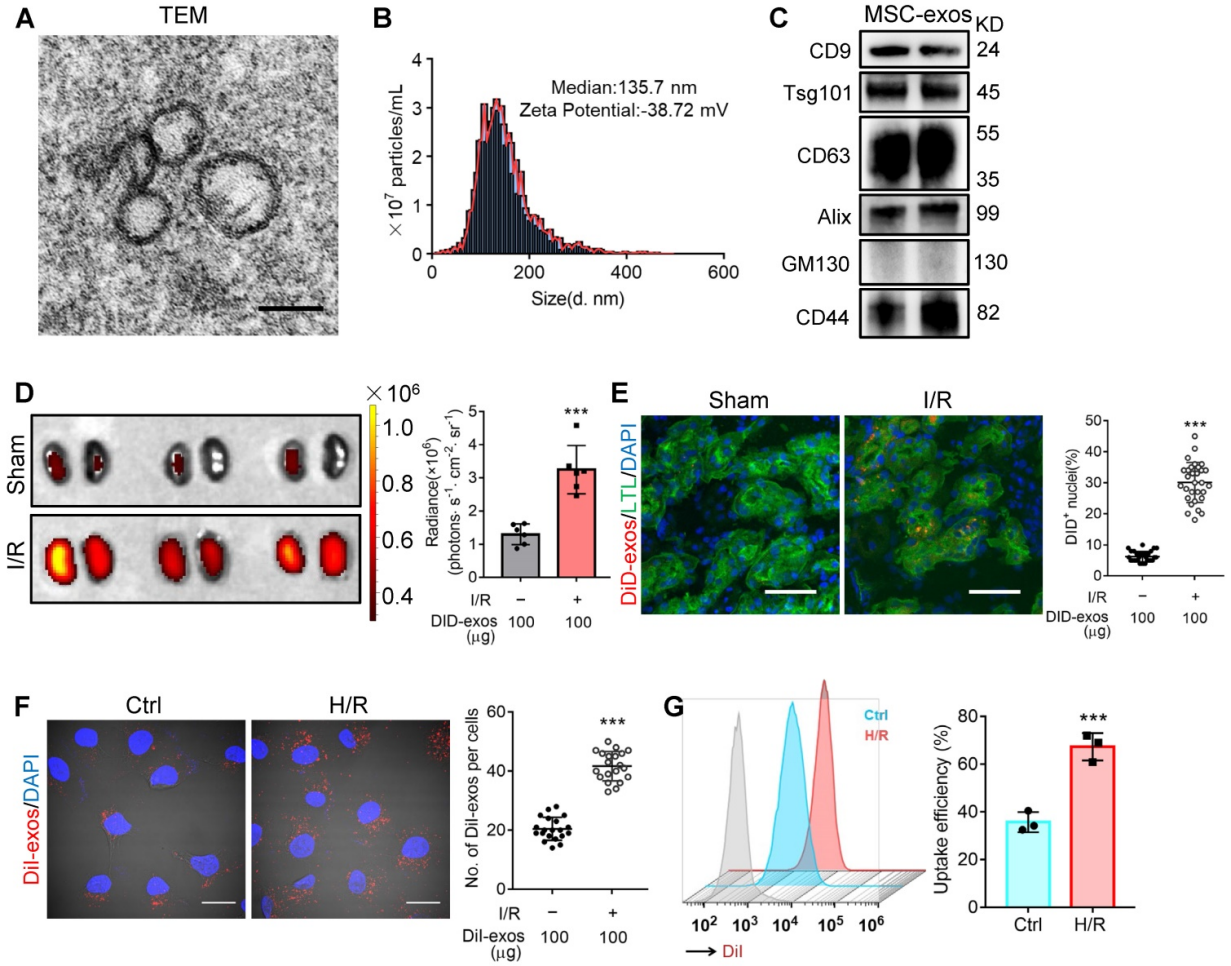
** MSC-exos homing to injured kidney.** (A) Morphology of MSC-exos under TEM. Scale bars, 100 nm. (B) Representative nanoparticle tracking analysis of MSC-exos. (C) Western blotting analysis of exosomal-associated (CD9, CD63, Tsg101, and Alix), Golgi-associated (GM130), and MSC-associated (CD44) markers in MSC-exos. (D) Imaging of fluorescence intensity in sham and I/R kidneys (n = 6). (E) Fluorescent images showed the accumulation of DiD-labeled MSC-exos in renal tubules of I/R mice. Scale bars, 25 μm. (F) Representative fluorescent images showed the uptake of DiI-labeled MSC-exos by H/R-treated HK-2 cells. Scale bars, 10 μm. (G) DiI-positive HK-2 cells were detected by flow cytometry. The blue peak represented the control and the red one represented H/R-treated HK-2 cells internalizing DiI-labeled MSC-exos. Data are presented as mean ± SD, ***p < 0.001, unpaired two-tailed Student's *t*-test.

Cultured hucMSCs were identified by optical microscopy (Figure S1A) and flow cytometry analysis (Figure S1B). The osteogenic, adipogenic and chondrogenic differentiation of hucMSCs was evaluated by Alizarin Red S, Oil Red O, and Alcian Blue/Nuclear Fast Red staining, respectively (Figure S1C). Next, the purified MSC-exos were characterized. TEM showed typical bilayer membrane vesicles (Figure 1A). NTA analysis indicated the median diameter of MSC-exos was 134.4 ± 3.9 nm (n = 4). The zeta potential was -37.93 ± 1.62 mV, indicating that MSC-exos were negatively charged and had high colloid stability (Figure 1B). MSC-exos expressed both exosome-associated (CD9, Tsg101, CD63, Alix) and MSC-associated protein (CD44), while the expression of Golgi marker (GM130) was not detectable (Figure 1C).

The biodistribution of MSC-exos was investigated by labeling exosomes with DiD. We intravenously injected DiD-labeled MSC-exos into sham or I/R injured mice. *Ex vivo* imaging of the dissected organs showed that DiD fluorescence was mainly distributed in the liver of sham mice (Figure S2). The renal radiance signals of mice with I/R-induced AKI were significantly higher compared with sham mice, as shown in Figure 1D. The fluorescence levels of the frozen kidney section also corroborated robust amounts of MSC-exos accumulating in LTL^+^ proximal tubules of I/R mice whereas very few in sham mice (Figure 1E). These findings indicated that MSC-exos exhibited preferential tropism to the injured kidneys which were predominantly internalized by proximal tubules.

Next, we examined the uptake of MSC-exos by renal TECs *in vitro*. DiI-labeled MSC-exos were incubated with HK-2 cells, with or without H/R injury. An increase of internalization of DiI-labeled MSC-exos was detected in H/R injured cells (Figure 1F). The uptake efficiency was approximately 67% of injured cells versus 36% of control cells at 6 h, as detected by flow cytometry (Figure 1G). To validate that MSC-exos were actively internalized by HK-2 cells, cells were incubated with DiI-labeled MSC-exos at 4 °C to eliminate endocytic trafficking (Figure S3). The results showed that DiI-labeled MSC-exos internalized by HK-2 cells at 4 °C were much less than 37 °C. These results suggested that MSC-exos exhibited preferential targeting to H/R injured TECs.

### Integrin VLA-4 and LFA-1 guide the homing of MSC-exos to injured kidney

**Figure 2:**
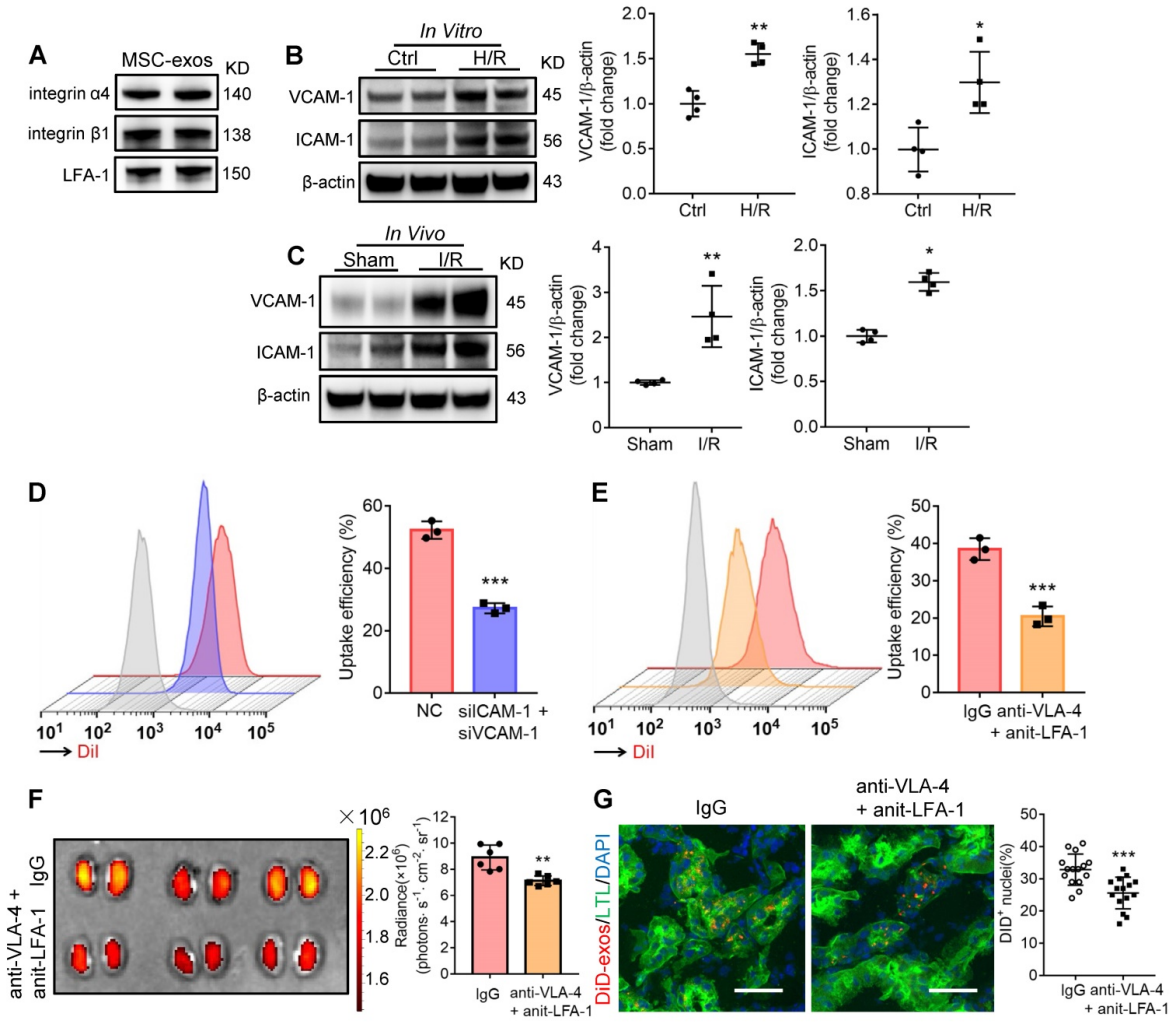
** Integrin VLA-4 and LFA-1 guide the homing of MSC-exos to injured kidney.** (A) Western blotting analysis of VLA-4 (integrin α4, integrin β1) and LFA-1 on MSC-exos. (B) Western blotting analysis of VCAM-1 and ICAM-1 in control or cells with H/R injury (n = 4). (C) Western blotting analysis of VCAM-1 and ICAM-1 in sham or I/R injured mice tissues (n = 4). (D) Flow cytometry detected the effect of siICAM-1 and siVCAM-1 transfection on cellular uptake of DiI-labeled MSC-exos in H/R-injured HK-2 cells. The red peak represented NC siRNA treated and the purple one represented siICAM-1 and siVCAM-1 transfected cells. (E) Flow cytometry detected the effect of VLA-4 and LFA-1 blockade on cellular uptake of DiI-labeled MSC-exos in H/R-injured HK-2 cells. The red peak represented isotype control treated MSC-exos and the orange one represented anti-VLA-4 and anti-LFA-1 antibodies treated MSC-exos. (F) Imaging of fluorescence intensity in isotype control or blocking antibodies treated group (n = 6). (G) Fluorescent images showed the accumulation of isotype control or blocking antibodies treated DiD-labeled MSC-exos in I/R mice (n = 6). Scale bars, 25 μm. Data are presented as mean ± SD, *p < 0.05, **p < 0.01, ***p < 0.001, unpaired two-tailed Student's *t*-test.

Generally, the membrane proteins of EVs determine their specific cellular interaction. Previous studies have shown that the interaction between very late antigen 4 (VLA-4; integrin-α4β1) and vascular cell adhesion molecule 1 (VCAM-1), or lymphocyte function-associated antigen 1 (LFA-1; integrin-αLβ2) and intercellular cell adhesion molecule-1 (ICAM-1) could mediate EVs adhesive to inflamed kidney 24. Indeed, we found that these integrins (integrin α4, integrin β1, LFA-1) were present on MSC-exos as indicated by western blotting (Figure 2A). Therefore, we assumed that VLA-4 and LFA-1 could guide the homing of MSC-exos to injured kidney, in which VCAM-1 and ICAM-1 were increased during I/R injury both *in vitro* and* in vivo* (Figure 2B-C). The down-regulation of ICAM-1 and VCAM-1 in H/R injured HK-2 cells markedly reduced the uptake of DiI-labeled MSC-exos (Figure 2D). In addition, we incubated DiI-labeled MSC-exos with blocking antibodies against VLA-4 and LFA-1 or with the corresponding isotype control, and found that the neutralization of VLA-4 and LFA-1 significantly reduced the accumulation of MSC-exos in H/R treated HK-2 cells (Figure 2E). Furthermore, the accumulation of DiD-labeled MSC-exos treated with anti-VLA-4 and anti-LFA-1 antibodies were also decreased in kidneys of I/R-induced AKI mice (Figure 2F-G), indicating VLA-4 and LFA-1 present on MSC-exos were responsible for their homing to injured kidneys.

### MSC-exos attenuate renal I/R injury in mice

**Figure 3:**
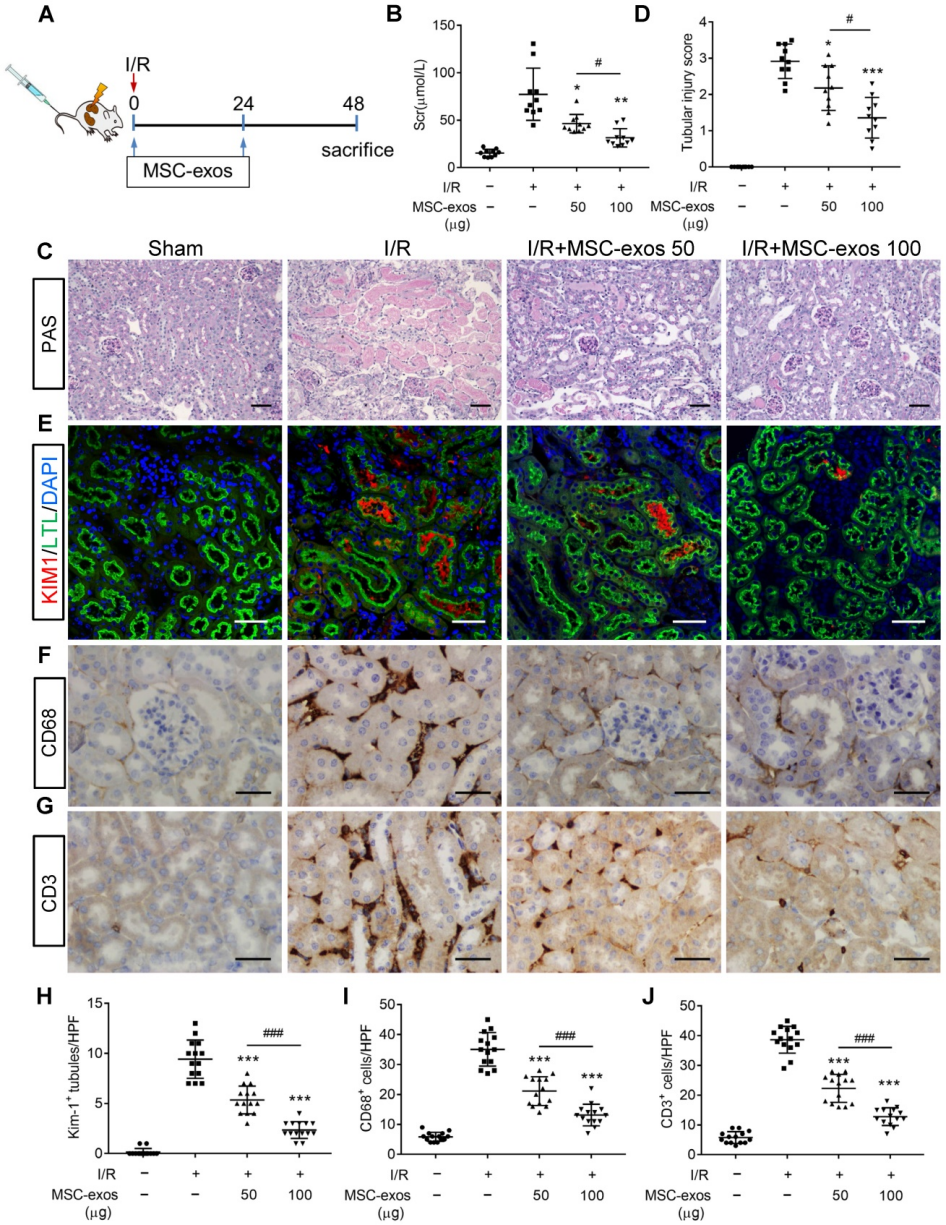
** MSC-exos attenuate renal I/R injury in mice.** (A) Schematic illustration of the experimental design. Briefly, mice were administered with MSC-exos (50 μg or 100 μg) or PBS at 0 h and 24 h after induction of renal I/R injury and euthanized at 48 h. (B) Dose-dependent effects of MSC-exos on serum creatinine (n = 10). (C) Representative images of renal I/R injuries by PAS staining. Scale bars, 50 μm. (D) The semi-quantification of tubular injury (n = 10). (E) Representative confocal images of KIM-1^+^ positive tubules. Scale bars, 25 μm. (F) Representative images of CD68^+^ macrophages in the tubulointerstitium. Scale bars, 50 µm. (G) Representative images of CD3^+^ T cells in the tubulointerstitium. Scale bars, 50 µm. (H) Quantification of KIM-1^+^ tubules per HPF (magnified 400×) (n = 7). HPF indicates high power field. (I) Quantification of CD68+ macrophages per HPF (magnified 400×) (n = 7). (J) Quantification of CD3+ T cells per HPF (magnified 400×) (n = 7). Data are presented as mean ± SD, *p < 0.05, **p < 0.01, ***p < 0.001 vs. I/R group, ^#^*p* < 0.05, ^###^*p* < 0.001, one-way ANOVA.

To explore the therapeutic effect of MSC-exos in I/R-induced AKI, the mice were injected intravenously with different doses of MSC-exos (50 μg or 100 μg), or PBS (n = 10, respectively) at 0 h and 24 h after renal reperfusion (Figure 3A). Bilateral I/R injury resulted in a noticeable elevation of serum creatinine (Scr), extensive swelling, vacuolar degeneration, necrosis and detachment of epithelial cell, and casts formation, which were attenuated by MSC-exos treatment in a dose-dependent manner (Figure 3B-D).

To test the protective feature of MSC-exos on renal tubules, KIM-1 (a tubule injury marker 32) was detected. The results indicated that KIM-1 was mainly expressed in LTL^+^ proximal tubules. The expression of KIM-1 was reduced by MSC-exos treatment in a dose-dependent manner (Figure 3E, 3H). The interstitial infiltration of inflammatory cells, including macrophages (CD68^+^) and T cells (CD3^+^), was markedly inhibited in MSC-exos-treated groups (Figure 3F, G, I, J). In addition, the mRNA expressions of TNF-α, IL-6, IL-1β, and monocyte chemotactic protein 1 (MCP-1) were also decreased by MSC-exos treatment (Figure S4). Collectively, these results suggested that MSC-exos could alleviate murine ischemic AKI and mitigate the proximal tubular injuries.

### MSC-exos rescue G2/M arrest and promote the proliferation of TECs

**Figure 4:**
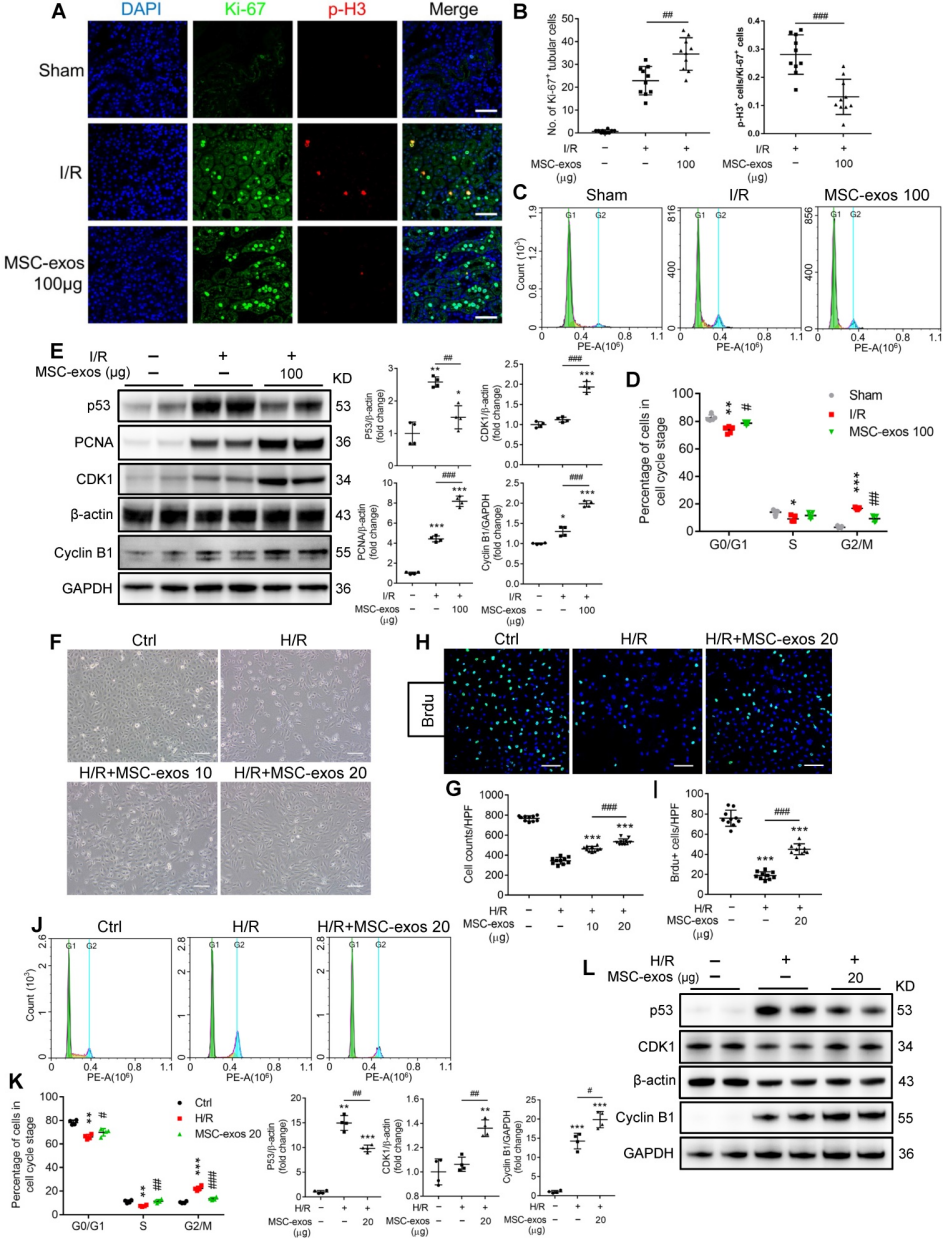
** MSC-exos rescue G2/M arrest and promote proliferation of TECs *in vivo* and *in vitro*.** (A) Representative confocal images of Ki-67^+^ and p-H3^+^ cells. Scale bars, 50 µm. (B) The quantification of Ki-67^+^ TECs and the proportion of p-H3^+^ cells/Ki-67^+^ cells (n = 5). (C) The cell cycle distribution of renal tubular cell suspension. (D) The proportion of renal tubular cells in different phases of cell cycle (n = 5). (E) Western blotting analysis of p53, PCNA, CDK1, and Cyclin B1 in kidney tissues (n = 4). (F) Representative optical micrograph of HK-2 cells in H/R and MSC-exos treated group. Scale bars, 25 µm. (G) Quantification of HK-2 cell counts per HPF (magnified 400×) (n = 10). (H) Representative images of Brdu+ cells. Scale bars, 100 µm. (I) Quantification of Brdu^+^ cells per HPF (magnified 400×) (n = 10). (J) The cell cycle distribution of HK-2 cells in different treatments. (K) The percentage of HK-2 cells in different phase of cell cycle (n = 4). (L) Western blotting of p53, CDK1, and Cyclin B1 in HK-2 cells (n = 4). Data are presented as mean ± SD, *p < 0.05, **p < 0.01, ***p < 0.001 vs. sham group or control group, ^#^*p* < 0.05,^ ##^*p* < 0.01, ^###^*p* < 0.001, two-tailed independent Student's *t*-test or one-way ANOVA.

After ischemic AKI, the cellular hallmark of renal repair is the prominent proliferation of surviving TECs. The proliferative TECs are partially suffered from cell cycle arrest in the G2/M phase 33. To investigate the effects of MSC-exos on cell cycle and proliferation of TECs, we labeled proliferating cells with Ki-67 and cells in G2/M phase with p-H3 staining. As shown in Figure 4A, the proportion of Ki-67^+^ cells in sham mice was less than 1%. At 48 h post-I/R injury, the proliferative cells of TECs (Ki-67^+^) increased, and the percentage of cells in G2/M phase (p-H3^+^) was approximately 28%. The treatment of MSC-exos significantly promoted the proliferation of TECs and decreased the percentage of cells in G2/M phase to approximately 13% (Figure 4A-B). Furthermore, we isolated the single-cell suspension of renal tubules and detected the cell cycle distribution by flow cytometry. The proportion of cell arrest in G2/M phase was less than 3% in sham mice, but it was increased to approximately 16% in I/R mice and was reduced to approximately 9% after treatment with MSC-exos (Figure 4C-D). Meanwhile, MSC-exos significantly suppressed the expression of cell cycle regulatory proteins p53, a key transcription factor participating in the G2 checkpoint by inhibiting cyclin-dependent kinases. The expressions of cyclin-dependent kinases 1 (CDK1) and Cyclin B1 were distinctly up-regulated by MSC-exos (Figure 4E).

To investigate whether MSC-exos could rescue G2/M arrest and promote the proliferation of TECs *ex vivo*, we conducted studies on two tubular cell lines: human HK-2 cells and mTECs. In HK-2 cells, H/R treatment resulted in a noticeable decrease of cell proliferation and increase cell arrest in G2/M phase, which were distinctly ameliorated by MSC-exos treatment (Figure 4F-K, Figure S5A). H/R injury significantly increased the proportion of G2/M phase to approximately 22%, and the proportion decreased close to 14% after MSC-exos treatment. Similar findings were also observed in mTECs (Figure S5B-C). In addition, MSC-exos significantly suppressed the expression of p53 and up-regulated the expressions of CDK1 and Cyclin B1 *in vitro* (Figure 4L). Taken together, MSC-exos showed the capacity to rescue TECs from G2/M arrest and promote TECs proliferation in I/R-induced AKI mice and *in vitro*.

### MSC-exos protect TECs from apoptosis induced by I/R injury

**Figure 5:**
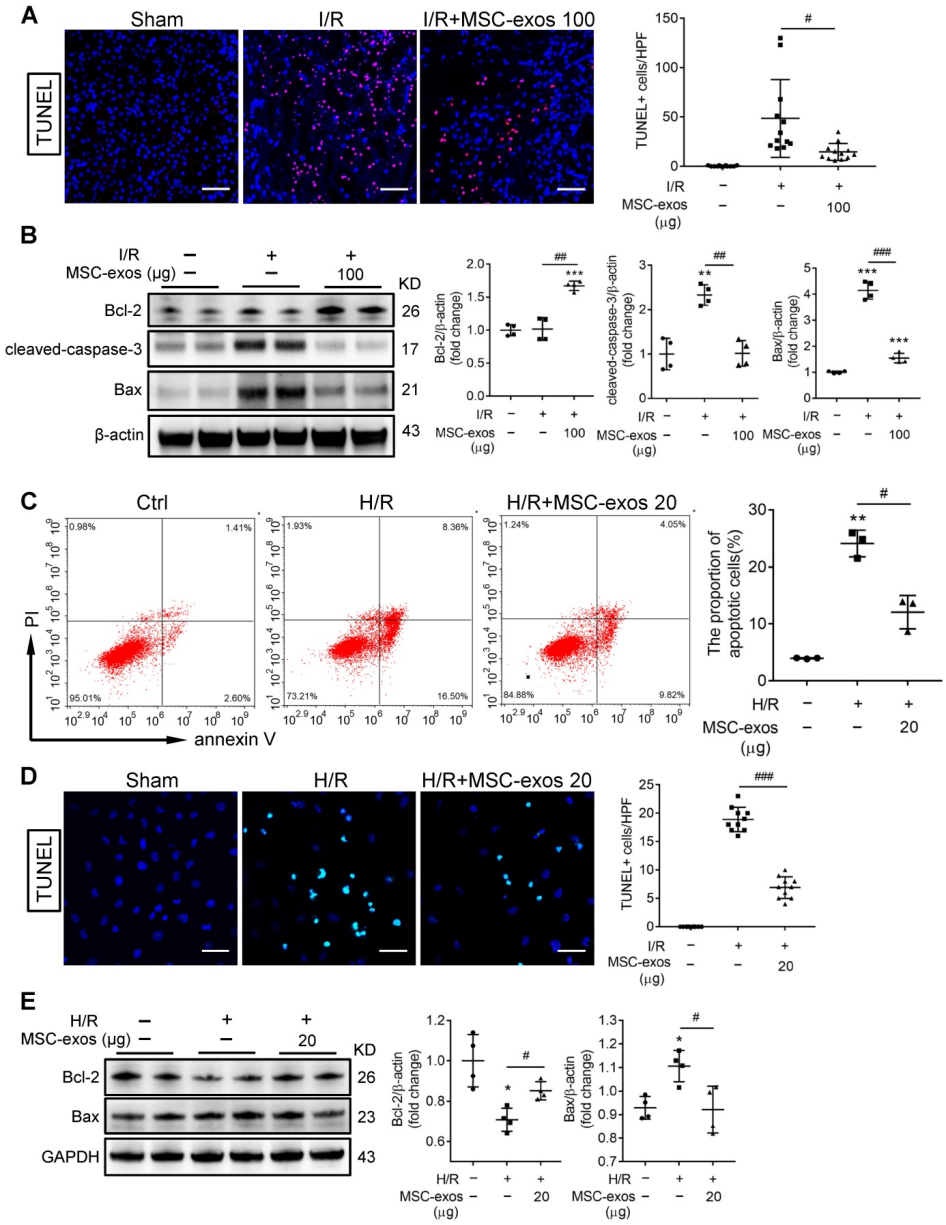
** MSC-exos protect TECs from apoptosis induced by I/R injury.** (A) Representative confocal images of TUNEL staining in kidney tissues and quantification of the apoptotic cells (n = 6). Scale bars, 50 μm. (B) Western blotting of Bcl-2, cleaved-caspase-3, and Bax in kidney tissues (n = 4). (C) Flow cytometry analysis of annexin V/PI staining and quantification of the apoptotic cells (n = 3). (D) Representative images of TUNEL staining in HK-2 cells and quantification of the apoptotic cells per HPF (magnified 400×) (n = 10). Scale bars, 50 µm. (E) Western blotting analysis of Bax and Bcl-2 in HK-2 cells (n = 4). Data are presented as mean ± SD, *p < 0.05, **p < 0.01, ***p < 0.001 vs. sham group or control group, ^#^*p* < 0.05,^ ##^*p* < 0.01, ^###^*p* < 0.001, one-way ANOVA.

During AKI, multiple forms of cell death including apoptosis and regulated necrosis contribute to kidney injury 34, 35. We next examined whether MSC-exos could ameliorate apoptosis of TECs during ischemic injury. As shown in Figure 5A, the apoptosis of TECs was induced by I/R injury, while markedly inhibited after MSC-exos administration, accompanied by decreasing expressions of apoptosis-related proteins Bax and cleaved-caspase-3, and up-regulation of anti-apoptosis protein Bcl-2 (Figure 5B).

Similarly, MSC-exos markedly reduced H/R-induced apoptosis of HK-2 cells*,* examined by annexin V/PI (Figure 5C) and TUNEL staining (Figure 5D). Moreover, MSC-exos inhibited the expression of Bax and up-regulated Bcl-2 (Figure 5E). Thus, our data showed MSC-exos effectively protected TECs from apoptosis induced by ischemic injury.

### miR-125b-5p is enriched in MSC-exos and delivers to TECs

**Figure 6:**
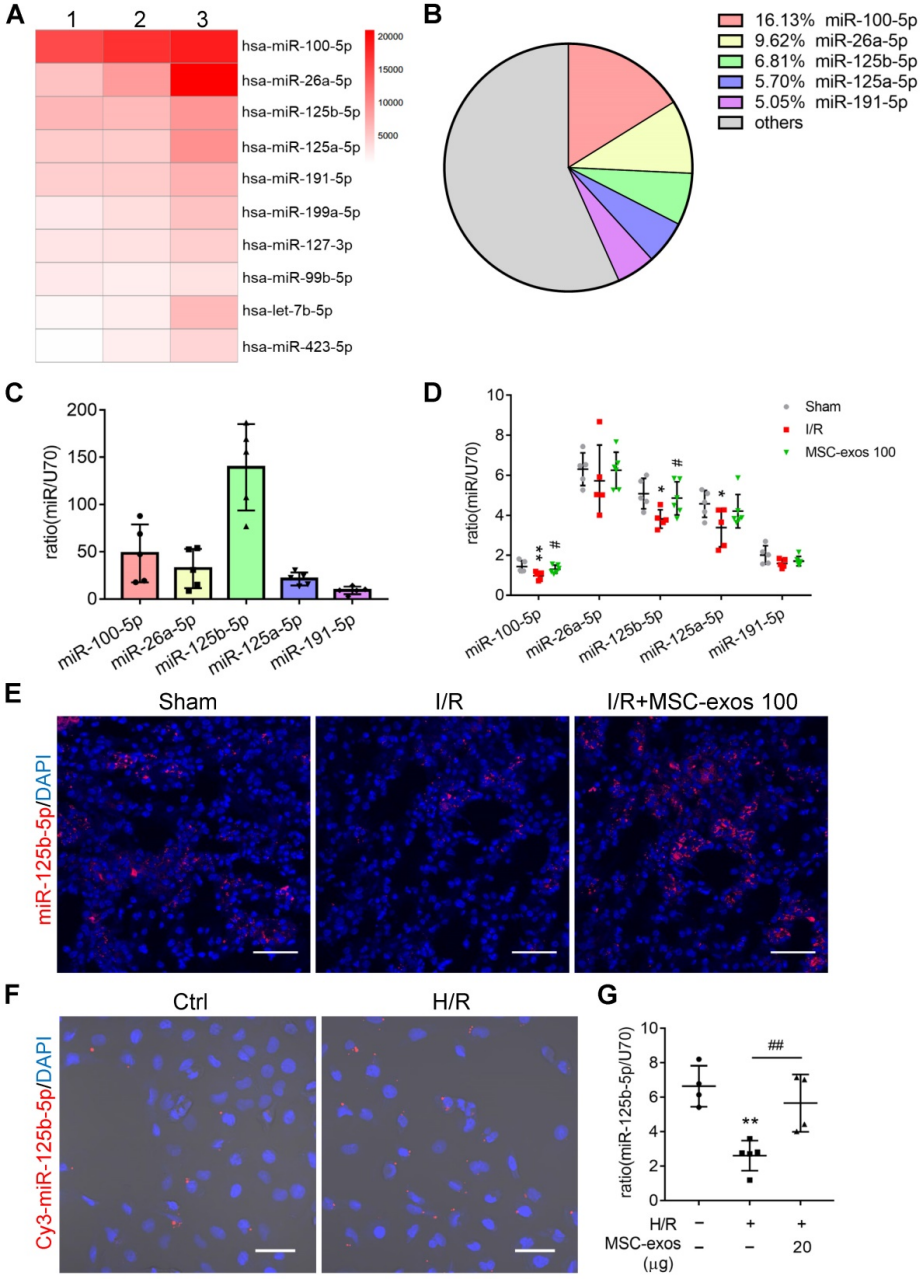
** miR-125b-5p is enriched in MSC-exos and delivers to TECs.** (A) Heat map of the top ten most abundant miRNAs in MSC-exos by miRNA-seq. (B) Relative percentage of miRNAs in total miRNA reads. (C) RT-PCR analysis of the top five most abundant miRNAs in MSC-exos (n = 5). (D) RT-PCR analysis of the top five miRNAs in MSC-exos-treated mice renal tissues (n = 5-6). (E) FISH analysis of miR-125b-5p in kidney tissues. Scale bars, 50 µm. (F) Representative images of Cy3-miR-125b-5p mimic-MSC-exos internalized by HK-2 cells. Scale bars, 50 µm. (G) RT-PCR analysis of miR-125b-5p in HK-2 cells (n = 4-5). Data are presented as mean ± SD, *p < 0.05, **p < 0.01 vs. sham group or control group, ^#^*p* < 0.05,^ ##^*p* < 0.01 vs. I/R or H/R group, one-way ANOVA.

To identify the miRNA expression profiles and the mechanism responsible for the therapeutic effects of MSC-exos, exosomal miRNAs were screened by miRNA-seq. It is notable that the top five most abundant miRNAs, including miR-100-5p, miR-26a-5p, miR-125b-5p, miR-125a-5p, and miR-191-5p, accounted for approximately 43% of the total miRNA reads (Figure 6A-B). Among them, the expression of miR-125b-5p was the highest in MSC-exos determined by RT-PCR (Figure 6C).

Next, we further measured the expression of the top five miRNAs in MSC-exos treated mice renal tissues. The treatment of MSC-exos significantly increased the expression of miR-100-5p and miR-125b-5p (Figure 6D). Furthermore, miR-125b-5p was detected in kidney tissues by FISH, which showed that MSC-exos treatment significantly increased miR-125b-5p expression in renal tubules (Figure 6E). To confirm miR-125b-5p could be delivered to H/R injured HK-2 cells by MSC-exos treatment, hucMSCs were transfected with the Cy3 labeled miR-125b-5p mimic, then the Cy3-miR-125b-5p mimic-MSC-exos were purified and incubated with HK-2 cells. The red fluorescence was clearly detected in HK-2 cells (Figure 6F). The expression of miR-125b-5p was up-regulated in H/R injured HK-2 cells by MSC-exos treatment determined by RT-PCR (Figure 6G). These results suggested that exosomal miR-125b-5p could be delivered to TECs.

### MSC-exos rescue G2/M arrest and reduce apoptosis via inhibiting p53 signaling through miR-125b-5p

**Figure 7:**
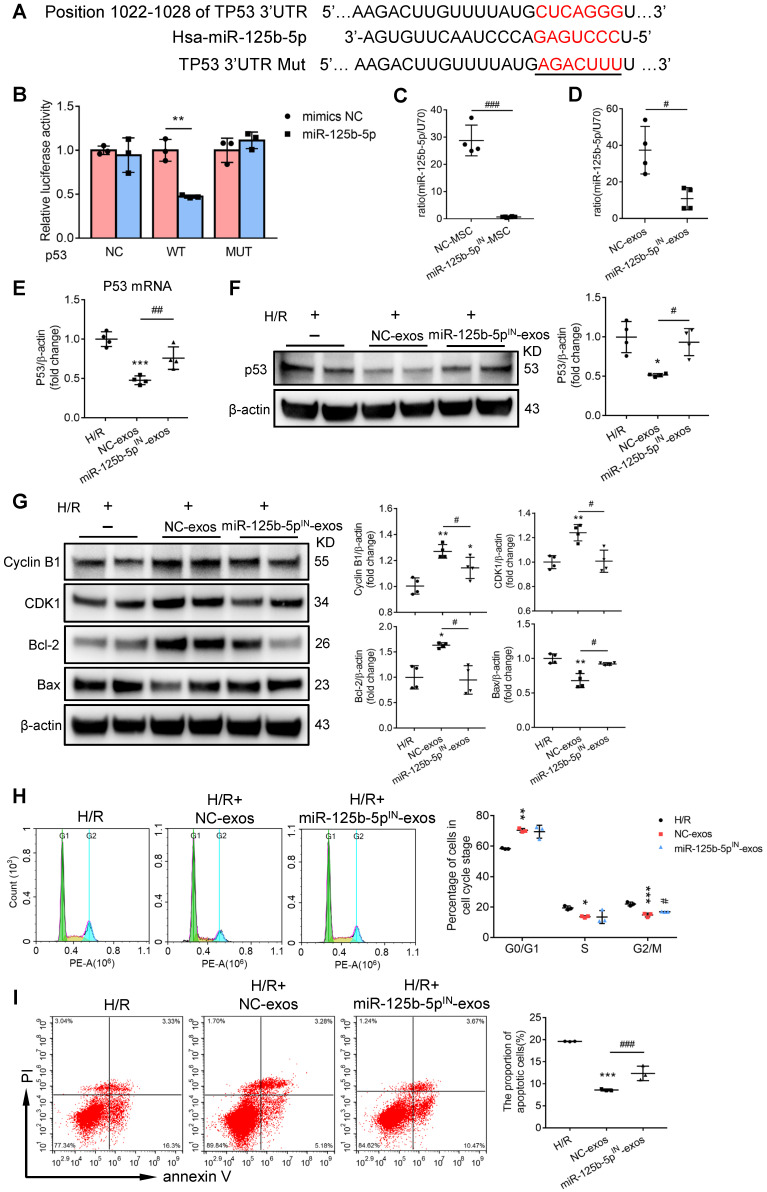
** MSC-exos rescue G2/M arrest and reduce apoptosis via inhibiting p53 signaling through miR-125b-5p.** (A) Schematic diagram depicted the predicted binding site of miR-125b-5p targeting the 3'-UTR of TP53. The underlined sequences represented the mutation seed sequence of 3'-UTR of TP53. (B) Luciferase reporter assay determined p53 as a bona fide target of miR-125b-5p (n = 3). NC, negative control; WT, wide type; MUT, mutant. (C) RT-PCR analysis of miR-125b-5p in miR-125b-5p inhibitor transfected MSCs (n = 4). (D) RT-PCR analysis of miR-125b-5p in exosomes isolated from miR-125b-5p inhibitor transfected MSC (miR-125b-5p^IN^-exos) (n = 4). RT-PCR (E) and Western blotting analysis (F) of p53 in HK-2 cells treated with miR-125b-5p^IN^-exos (n = 4). (G) Western blotting analysis of Cyclin B1, CDK1, Bcl-2 and Bax in HK-2 cells treated with miR-125b-5p^IN^-exos (n = 4). Flow cytometry analysis of cell cycle (H) and annexin V/PI staining (I) in miR-125b-5p^IN^-exos treated HK-2 cells (n = 3). Data are presented as mean ± SD, *p < 0.05, **p < 0.01, ***p < 0.001 vs. H/R group, ^#^*p* < 0.05,^ ##^*p* < 0.01, ^###^*p* < 0.001, unpaired Student's *t*-test (two-tailed) or one-way ANOVA.

To further investigate the exact effects of exosomal miR-125b-5p, we predicted the putative targets of miR-125b-5p which may participate in the regulation of proliferation and apoptosis from four online databases: microRNA.org, DIANA-TarBase, miRanda, and TargetScan. Overlap analysis revealed that p53 was the putative target of miR-125b-5p (Figure 7A). Notably, luciferase reporter assay demonstrated that the activity of luciferase reporters was clearly reduced by miR-125b-5p overexpression when compared with miRNA-NC. Furthermore, the activity of TP53-3′-UTR-mutant luciferase reporter was not affected by the miR-125b-5p overexpressed vector, suggesting that p53 was a bona fide target of miR-125b-5p (Figure 7B). p53 has been proven to play a crucial role in the pathogenesis of AKI through regulating various cellular biologic processes 36, 37. To prove that MSC-exos could rescue G2/M arrest and reduced apoptosis via inhibiting p53 signaling, we performed an overexpression experiment of p53 in HK-2 cells. As shown in Figure S6A, the efficiency of pEX-P53 was confirmed by increasing p53 protein expression. Compared with H/R+MSC-exos+pEX-1 group, the proportion of cells in the G2/M phase was increased in H/R+MSC-exos+pEX-P53 group (Figure S6B), as well as the proportion of apoptotic cells (Figure S6C). These results indicated that MSC-exos rescued G2/M arrest and reduced apoptosis via inhibiting p53 signaling.

To investigate whether exosomal miR-125b-5p was involved in the down-regulation of p53 in HK-2 cells, hucMSCs were transfected with miR-125b-5p inhibitor to construct miR-125b-5p-inhibitor transfected MSCs (miR-125b-5p^IN^-MSC) (Figure 7C). As shown in Figure 7D, the exosomes isolated from miR-125b-5p-inhibitor transfected MSCs (miR-125b-5p^IN^-exos) expressed less miR-125b-5p compared with the exosomes isolated from NC transfected MSCs (NC-exos). When NC-exos or miR-125b-5p^IN^-exos were administered to HK-2 cells treated with H/R, the result showed that the knockdown of miR-125b-5p markedly reversed MSC-exos induced down-regulation of p53 (Figure 7E-F). Furthermore, we examined the downstream protein levels of p53 involving in pro-apoptosis and cell cycle arrest. As shown in Figure 7G, miR-125b-5p^IN^-exos markedly reversed the decrease of Bax and the increase of Cyclin B1, CDK1, and Bcl-2 in NC-exos treatment group. Consistently, the therapeutic effects of rescuing G2/M phase arrest and inhibiting cell apoptosis were also decreased after miR-125b-5p^IN^-exos treatment when compared with NC-exos treated cells (Figure 7H-I). These results indicated that MSC-exos rescued G2/M arrest and reduced apoptosis via down-regulation of p53 signaling via miR-125b-5p.

### miR-125b-5p inhibitor mitigates the protective effects of MSC-exos in I/R mice

**Figure 8:**
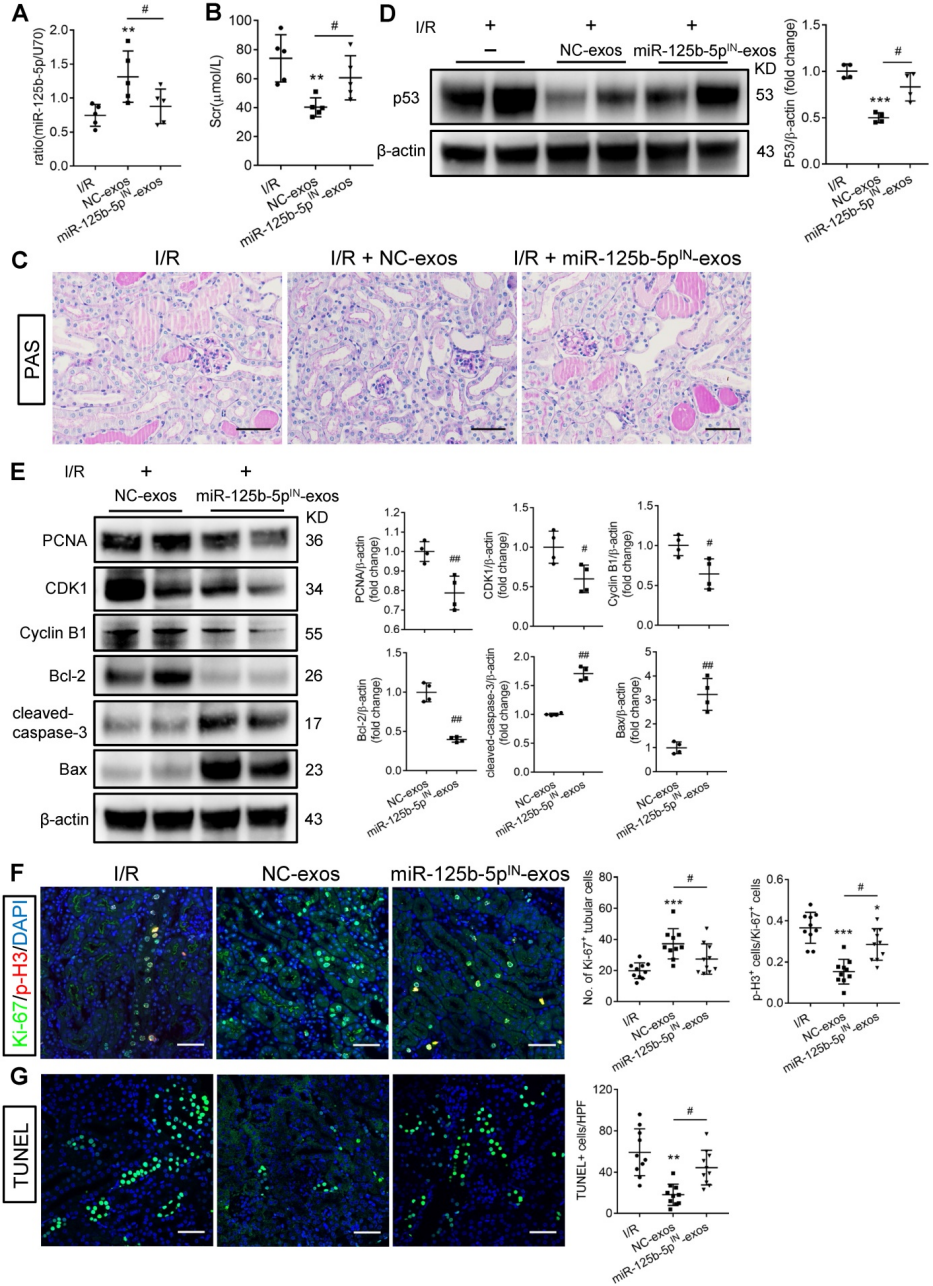
** miR-125b-5p inhibitor reduces the protective effects of MSC-exos in I/R mice.** (A) RT-PCR analysis of miR-125b-5p in NC-exos or miR-125b-5p^IN^-exos treated I/R mice (n = 5). (B) Effects of NC-exos or miR-125b-5p^IN^-exos on serum creatinine (n = 5). (C) Representative images of PAS staining. Scale bars, 50 µm. (D) Western blotting analysis of p53 in NC-exos or miR-125b-5p^IN^-exos treated I/R mice (n = 4). (E) Western blotting analysis of PCNA, Cyclin B1, CDK1, cleaved-caspase-3, Bcl-2 and Bax in NC-exos or miR-125b-5p^IN^-exos treated I/R mice (n = 4). (F) Representative confocal images of Ki-67^+^ (green) and p-H3^+^ (red) cells in kidney tissues (n = 5). Scale bars, 50 µm. (G) Representative confocal images of TUNEL staining in kidney tissues and quantification of the apoptotic cells (n = 5). Scale bars, 50 µm. Data are presented as mean ± SD, *p < 0.05, **p < 0.01, ***p < 0.001 vs. I/R group, ^#^*p* < 0.05,^ ##^*p* < 0.01, two-tailed unpaired Student's t-test or one-way ANOVA.

To investigate whether miR-125b-5p was involved in MSC-exos-mediated tubular repair *in vivo*, NC-exos or miR-125b-5p^IN^-exos were injected into I/R mice model. After treatment with miR-125b-5p^IN^-exos, the level of miR-125b-5p in renal tissues was not increased (Figure 8A). Mice treated with miR-125b-5p^IN^-exos showed higher Scr levels than those in the NC-exos group (Figure 8B). And the epithelial cell swelling, necrosis, cellular debris accumulation, and casts formation were noticeably observed in miR-125b-5p^IN^-exos-treated mice (Figure 8C). The knockdown of miR-125b-5p significantly reversed the inhibitory effect of NC-exos on p53 expression in mice kidneys (Figure 8D). The downstream molecules of p53, including CDK1, Cyclin B1, Bcl-2, and Bax, were also markedly reversed by miR-125b-5p^IN^-exos treatment (Figure 8E). We next examined the effects of miR-125b-5p^IN^-exos on the cell cycle and apoptosis of TECs. Compared with NC-exos-treated group, the Ki-67^+^ proliferative cells were decreased and the proportion of cells in G2/M phase was increased by miR-125b-5p^IN^-exos treatment (Figure 8F). The apoptotic cells were also markedly increased in miR-125b-5p^IN^-exos-treated group (Figure 8G). Overall, these results clearly demonstrated that MSC-exos were an effective therapy for ischemic AKI by miR-125b-5p/p53 pathway *in vivo*.

## Discussion

**Figure 9:**
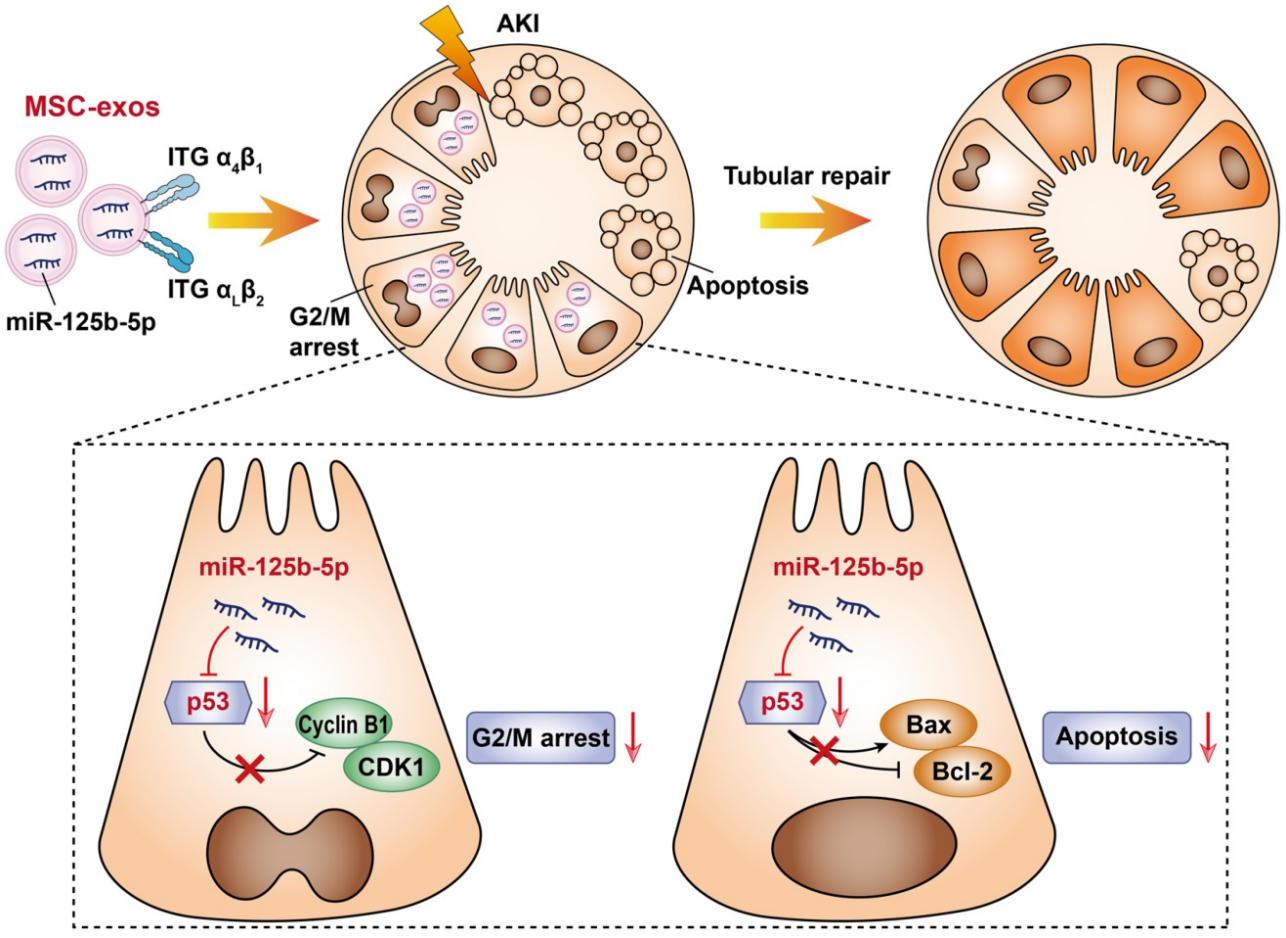
** Schematic illustration of the mechanism of MSC-exos for the treatment of ischemic AKI.** In ischemic AKI, the injuries of TECs could lead to cell cycle arrest in G2/M phase and apoptosis. MSC-exos targeted injured kidney especially the proximal tubules due to VLA-4 and LFA-1 mediated adhesive interactions. Moreover, miR-125b-5p was enriched in MSC-exos and exerted tubular repair effect via suppressing the expression of p53, which not only up-regulated CDK1 and Cyclin B1 to rescue tubular G2/M arrest, but modulated Bcl-2 and Bax to inhibit TECs apoptosis.

In this study, we assessed the effect of MSC-exos on promoting tubular repair in ischemic AKI. We found that MSC-exos exhibited preferential target to injured kidney and mainly accumulated in proximal tubules, owing to the adhesive components including VLA-4 and LFA-1 on the surface of exosomes. Importantly, we demonstrated for the first time that MSC-exos could attenuate G2/M arrest, promote proliferation and limit apoptosis of TECs via miR-125b-5p/p53 signaling pathway, and consequently promote renal repair after ischemic AKI (Figure 9).

Adult stem cells are widely used for the treatment of multiple human diseases by secreting soluble factors and EVs 38. MSCs exist in different tissues, including bone marrow, fat, umbilical cord, placenta tissue, and amnion. By far, the most prevalent source of MSCs in preclinical and clinical studies is bone marrow 39. However, the extraction of bone marrow is a highly invasive procedure, and the differentiation potential is declined with increasing age. By contrast, hucMSCs possess many desirable advantages, such as non-invasive, non-ethical controversy, low immunogenicity, easy to expand *in vitro* and prominent tissue repair properties 40,41. Moreover, hucMSCs improved GFR and tubular function better than adipose-derived MSCs in I/R-induced renal damage 42. The proteomics analysis showed that hucMSC-derived exosomes were more prominent in tissue damage repair than bone marrow MSCs (BM-MSCs) and adipose MSC-derived exosomes 43. Thus, we chose hucMSCs as the source of MSC-exos.

Firstly, we identified that MSC-exos exhibited preferential targeting to injured kidneys when injected through the tail vein and mainly accumulated in proximal tubules. Unlike most MSCs passively homing to the lung 44, nano-scale MSC-exos prioritized accumulation in mononuclear phagocytic system organs (liver, lungs, and spleen) in the sham mice and the accumulation in the kidney was extremely scarce. However, the distribution was altered in ischemic AKI mice. Similarly, Grange et al. 45 investigated the biodistribution of EVs in glycerol-induced AKI mice, and found EVs were only detectable in the injured kidneys. However, only a small number of systemically administered MSC localized within the injured tubules 46. Moreover, the poor survival of MSC from the injured renal tissues supported that MSC-exos may represent a promising therapeutic strategy for tubule repair after AKI. Generally, the membrane proteins present on EVs allow their targeting and capture by recipient cells. Our previous studies demonstrated that VLA-4 and LFA-1 on the surface of macrophage-derived microvesicles enabled them to adhere to the injured kidney through integrin-mediated adhesive interactions (VLA-4/VCAM-1 and LFA-1/ICAM-1) 24, 47. In this study, we found VLA-4 and LFA-1 were expressed on MSC-exos. Meanwhile, VCAM-1 and ICAM-1 were indeed overexpressed in I/R injured renal tissues and H/R injured HK-2 cells. The knockdown of ICAM and VCAM in HK-2 cells, or blockade of VLA-4 and LFA-1 on MSC-exos could reduce its uptake by TECs. Thus, we propose that MSC-exos naturally target injured kidney, especially the TECs with the favor of these unique integrin interactions.

TECs are metabolically active and exquisitely sensitive to hypoxia, septic, or toxic compounds, and the injury or death of TECs is the most common pathological change of AKI with multiple etiologies 7, 35, 48. After acute injury, surviving TECs could exhibit a proliferative phenotype, but partly proliferative TECs are suffered from cell cycle arrest 6, 49. Yang et al. 33 demonstrated that epithelial cell cycle G2/M arrest mediated chronic fibrotic kidney disease after AKI through activating c-jun NH_2_-terminal kinase signaling and up-regulation of profibrotic cytokines. Since then, compelling evidence has shown that sustained cell cycle G2/M arrest of TECs shows a profibrotic secretory phenotype, cell senescence, and maladaptive repair 50. In mice with unilateral I/R injury, abrogating the G2/M arrest could rescue fibrosis in ischemic injured kidney 33. Rescuing TECs from G2/M arrest may become a pivotal target for AKI treatment. In addition, the apoptosis of TECs is ubiquitous in AKI. In the present study, we found that MSC-exos are capable of promoting renal tubule repair through targeting the critical pathological events of AKI, including cell cycle arrest as well as apoptosis of TECs.

Next, we explored that miR-125b-5p enriched in MSC-exos and exerted a critical tubular protective effect via rescuing G2/M arrest and reducing apoptosis of TECs. miR-125b-5p, located on chromosome 11q24 and homologue with nematode lin-4, was considered as an anti-apoptotic miRNA 51, 52.Zhu et al. 52 found that the exosomes secreted from hypoxia-elicited BM-MSCs enriched of miR-125b-5p facilitated cardiac repair by ameliorating cardiomyocyte apoptosis. In this study, we observed that miR-125b-5p inhibitor decreased the protective effects of MSC-exos in I/R mice, which confirmed the renal repair effect of miR-125b-5p. However, it cannot be excluded that other miRNAs or other targets of miR-125b-5p may also have protective effects. The role of other exosomal miRNAs in renal repair needs to be further clarified.

Finally, we further identified the potential role of miR-125b-5p/p53 pathway in promoting tubule repair. p53, a well-known tumor suppressor transcription factor, can be activated in response to cellular stress, such as hypoxia, reactive oxygen species, and DNA damage. It also plays a crucial role in the pathogenesis of AKI through regulating various cellular biologic processes, including cell cycle arrest, apoptosis, and autophagy 36, 37. Our studies and other studies have all shown that proximal tubules were the primary tubule fragments that suffered from ischemic injury during AKI 48. Interestingly, our study showed MSC-exos mainly accumulated in injured proximal tubules, suggesting that proximal tubules were the potential target for MSC-exos mediated tubular repair. Previous studies have shown that the activation of p53 in proximal tubular cells promoted AKI 37. p53-mediated TECs cycle arrest and apoptosis play a crucial role in AKI pathogenesis, and thus modulation of p53 may provide a novel therapeutic strategy for AKI. The suppressing of p53 could protect against TECs injury 53. Interestingly, a Phase 3 study with siRNA targeting p53 (QPI-1002) is undergoing to evaluate the prevention for AKI following cardiac surgery (https://clinicaltrials.gov/ct2/show/NCT03510897). Due to the distinct advantages of low immunogenicity, biocompatibility, biological barrier permeability, and stability 14,47,54, MSC-exos carrying naturally loaded miR-125b-5p may represent a superior therapeutic strategy of repressing p53 signaling for tubular repair in AKI.

## Conclusions

In summary, MSC-exos exhibited preferential tropism to injured kidney, especially proximal tubules by virtue of VLA-4 and LFA-1 mediated adhesive interactions. Furthermore, MSC-exos ameliorated ischemic AKI and promote tubular repair by targeting the cell cycle arrest and apoptosis of TECs through miR-125b-5p/p53 pathway. These data identified a novel mechanism of MSC-exos in renal repair which may indicate an exciting opportunity for developing the innovative therapeutic approach for tubular repair in ischemic AKI.

## Supplementary Material

Supplementary figures and tables.

## Abbreviations

MSC-exos: Mesenchymal stem cells-derived exosomes
AKI: Acute kidney injury
hucMSCs: Human umbilical cord mesenchymal stem cells
I/R: Ischemia/reperfusion
TECs: Tubular epithelial cells
MSCs: Mesenchymal stem cells
EVs: Extracellular vesicles
miRNA-seq: miRNA sequencing
FBS: Fetal bovine serum
PBS: Phosphate buffer saline
TEM: Transmission electron microscopy
NTA: Nanoparticle tracking analysis
DiD: 1,1'-dioctadecyl-3,3,3',3'-tetramethylindodicarbocyanine perchlorate
DiI: 1,1′-dioctadecyl-3,3,3′,3′-tetramethylindocarbocyanine perchlorate
DAPI: 4',6-diamidino-2-phenylindole
mTECs: mouse tubular epithelial cells
H/R: Hypoxia/reoxygenation
CCK-8: Cell counting kit-8
TUNEL: Terminal deoxynucleotidyl transferase mediated dUTP nick end labeling
PI: Propidium iodide
PAS: Periodic acid-Schiff
FISH: Fluorescence *in situ* hybridization
KIM-1: Kidney injury molecular-1
RT-PCR: real-time PCR
SD: Standard deviation
VLA-4: Very late antigen 4
VCAM-1: Vascular cell adhesion molecule 1
LFA-1: Lymphocyte function-associated antigen 1
ICAM-1: Intercellular cell adhesion molecule-1
Scr: Serum creatinine
MCP-1: Monocyte chemotactic protein 1
CDK1: Cyclin-dependent kinases 1
miR-125b-5p^IN^-MSC: miR-125b-5p-inhibitor transfected MSCs
miR-125b-5p^IN^-exos: exosomes isolated from miR-125b-5p-inhibitor transfected MSCs
NC-exos: exosomes isolated from NC transfected MSCs
BM-MSCs: bone marrow mesenchymal stem cells
