# Exosomal miR-125b-5p deriving from mesenchymal stem cells promotes tubular repair by suppression of p53 in ischemic acute kidney injury: Erratum

**DOI:** 10.7150/thno.79609

**Published:** 2024-05-15

**Authors:** Jing-Yuan Cao, Bin Wang, Tao-Tao Tang, Yi Wen, Zuo-Lin Li, Song-Tao Feng, Min Wu, Dan Liu, Di Yin, Kun-Ling Ma, Ri-Ning Tang, Qiu-Li Wu, Hui-Yao Lan, Lin-Li Lv, Bi-Cheng Liu

**Affiliations:** 1 Institute of Nephrology, Zhong Da Hospital, Southeast University School of Medicine, Nanjing 210009, China.; 2 Department of Medicine and Therapeutics, Li Ka Shing Institute of Health Sciences, Liu Che Woo Institute of Innovative Medicine, Chinese University of Hong Kong, Hong Kong SAR 999077, China.

The authors apologize that the original version of the above article contains errors that need to be corrected. An incorrect image for I/R group in Figure 6E was inserted in figure assembly. The authors declare that these amendments do not change the results or conclusions of their paper. The authors sincerely apologize to the Journal and its readers for the confusion this may have caused. The corrected version of Figure 6E appears below.

## Figures and Tables

**Figure 6:**
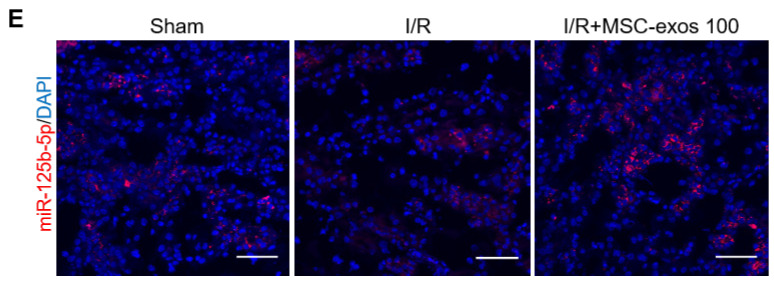
Corrected figure for original Figure 6E.

