# Supplementary material for: Exosomal miR-125b-5p deriving from mesenchymal stem cells promotes tubular repair by suppression of p53 in ischemic acute kidney injury
Source: Theranostics. 2021 Mar 11;11(11):5248–66. doi: 10.7150/thno.54550 (PMC8039965; doi:10.7150/thno.54550)
Supplement: Supplementary file 1 — Supplementary figures and tables. [file thnov11p5248s1.pdf]

Exosomal miR-125b-5p deriving from mesenchymal stem cells promotes tubular repair by suppression of p53 in ischemic acute kidney injury

Authors:

Jing-Yuan Cao*[1], Bin Wang*[1], Tao-Tao Tang*[1], Yi Wen[1], Zuo-Lin Li[1], Song-Tao Feng[1], Min Wu[1], Dan Liu[1], Di Yin[1], Kun-Ling Ma[1], Ri-Ning Tang[1], Qiu-Li Wu[1], Hui-Yao Lan[2], Lin-Li Lv[1], and Bi-Cheng Liu[1]

Supplementary figures and table

Figure S1

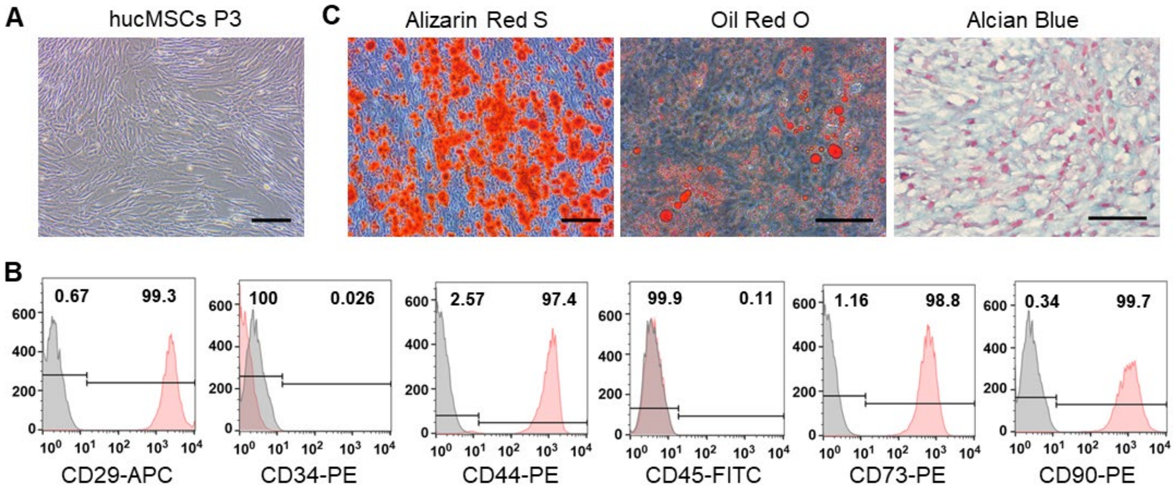

Figure S1. Identification of hucMSCs. **(**A) Representative optical micrograph of hucMSCs. Scale bars, 50 μm. (B) Alizarin Red S, Oil Red O, and Alcian Blue and Nuclear Fast Red staining of hucMSCs. Scale bars, 50 μm. (C) Flow cytometry analysis of MSCs associated surface markers (CD29, CD34, CD44, CD45, CD73 and CD90). Gray peaks represent the isotype controls and the red peaks represent the marker indicated.

Figure S2

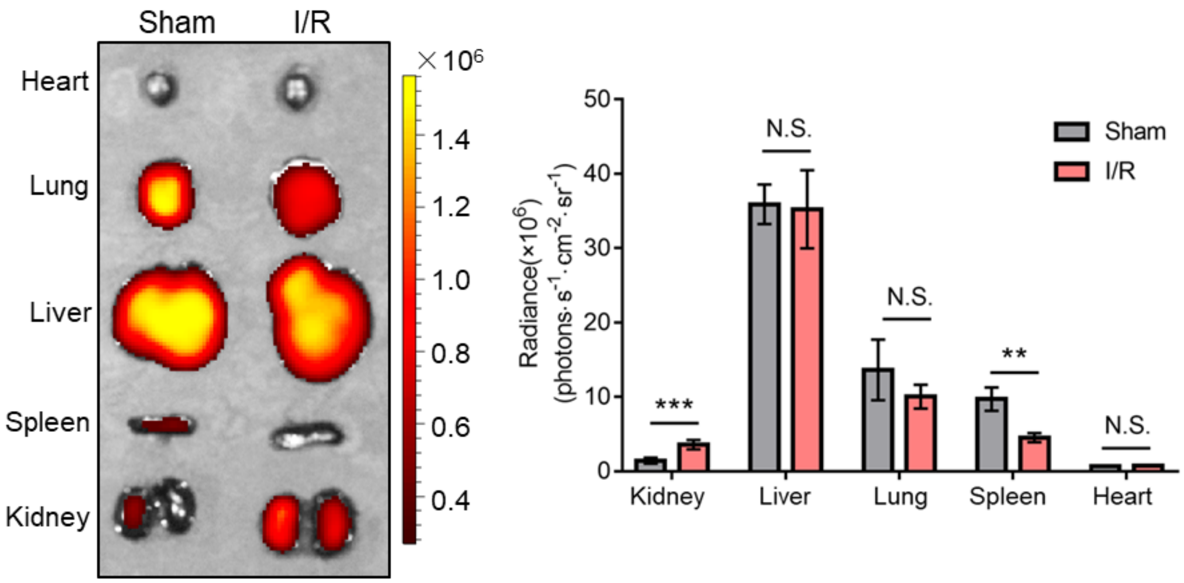

Figure S2. Imaging of fluorescence intensity at 12 h in sham and I/R mice (n = 3). $^{**}p < 0.01$, $^{***}p < 0.001$, N.S. $p > 0.05$.

Figure S3

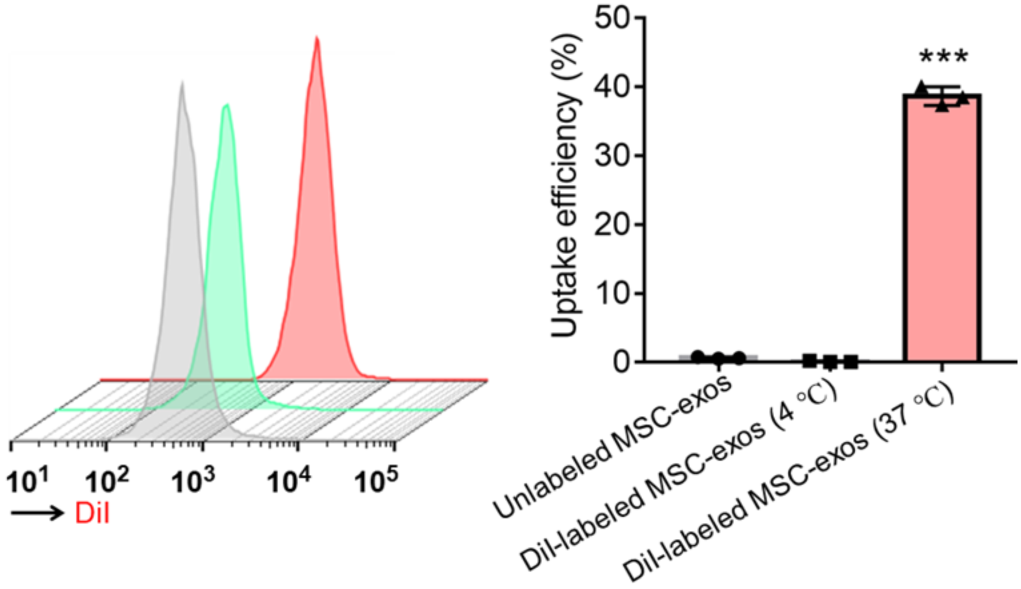

Figure S3. DiI-labeled MSC-exos uptake in standard culture medium conditions vs. in complete medium at 4 °C. ***$p < 0.001$.

Figure S4

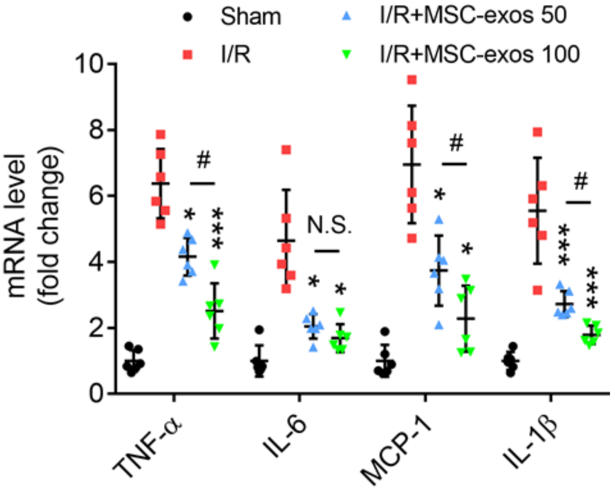

Figure S4. The anti-inflammatory efficacy of MSC-exos in I/R mice. RT-PCR analysis of mRNA levels of inflammatory cytokines in kidney tissues (n = 6). Data are presented as mean ± SD, *$p < 0.05$, ***$p < 0.001$ vs. I/R group, $^{\#}p < 0.05$, N.S. $p > 0.05$, one-way ANOVA.

Figure S5

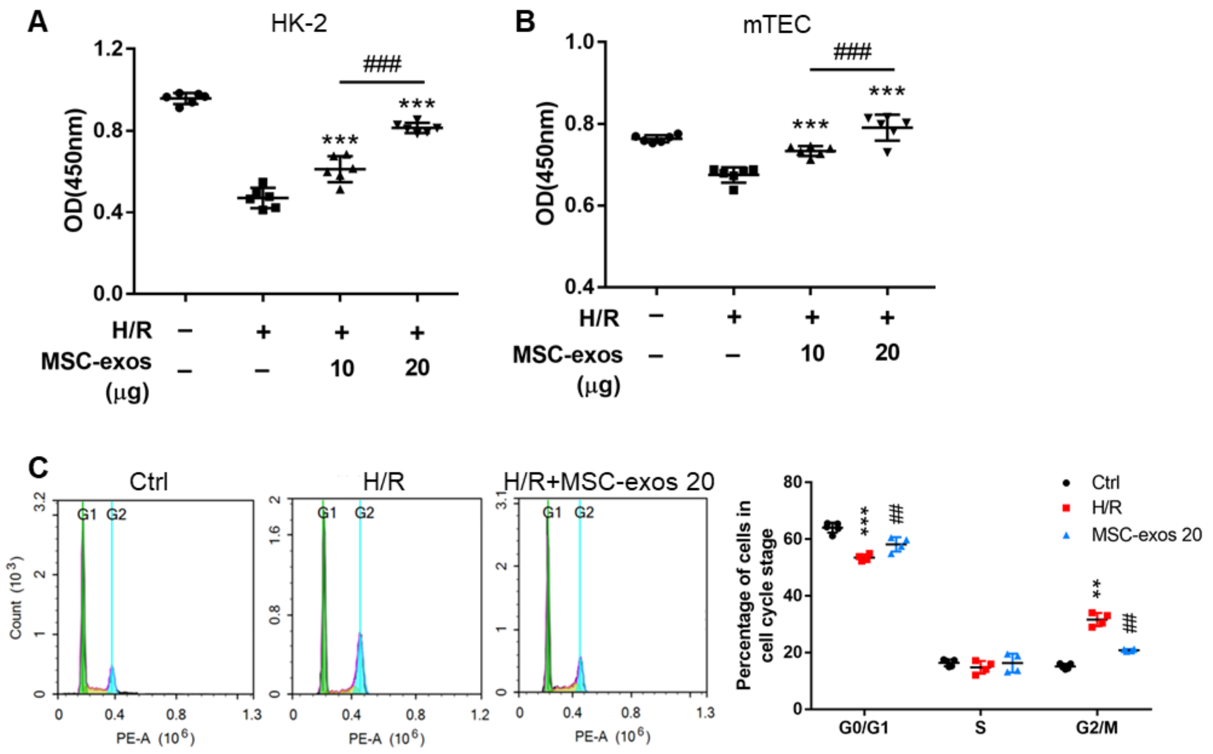

Figure S5. MSC-exos relieve G2/M arrest and promote proliferation of TECs *in vitro*. (A) CCK-8 assay in HK-2 cells (n = 6). ***p < 0.001 vs. I/R group, ###p < 0.001. (B) CCK-8 assay in mTECs (n = 6). ***p < 0.001 vs. I/R group, ###p < 0.001. (C) The cell cycle distribution of mTECs in different treatment. Data are presented as mean ± SD, **p < 0.01, ***p < 0.001 vs. control group, ##p < 0.01, one-way ANOVA.

Figure S6

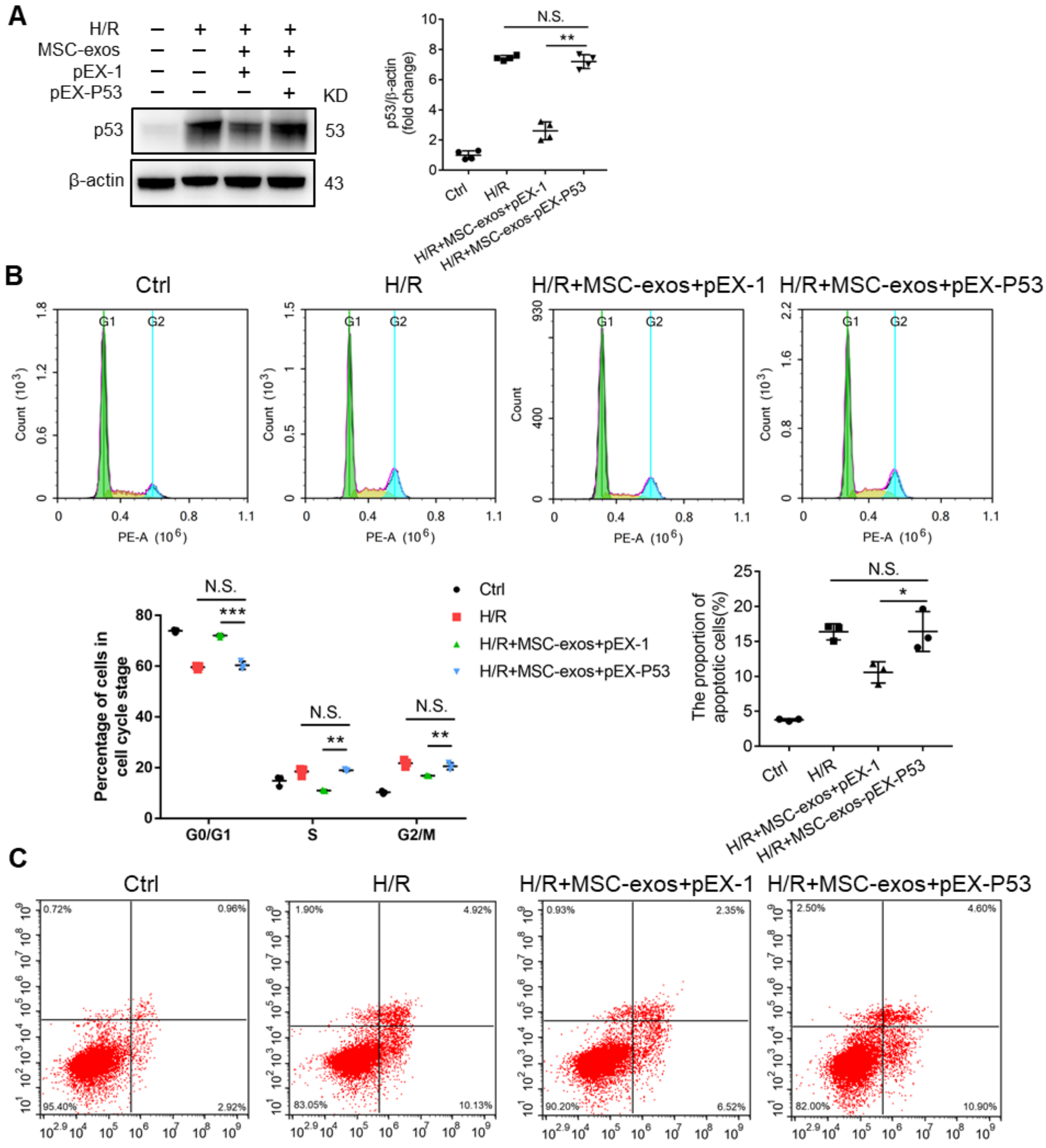

Figure S6. The overexpression of p53 reduces the therapeutic effect of MSC-exos. (A) Western blotting analysis of p53 in different treatments (n = 4). (B) The cell cycle distribution of HK-2 cells in different treatments (n = 3). (C) Flow cytometry analysis of annexin V/PI staining and quantification of the apoptotic cells (n = 3). Data are presented as mean ± SD, *$p < 0.05$, **$p < 0.01$, ***$p < 0.001$, one-way ANOVA.

Figure S7

Unedited images for figure 1C

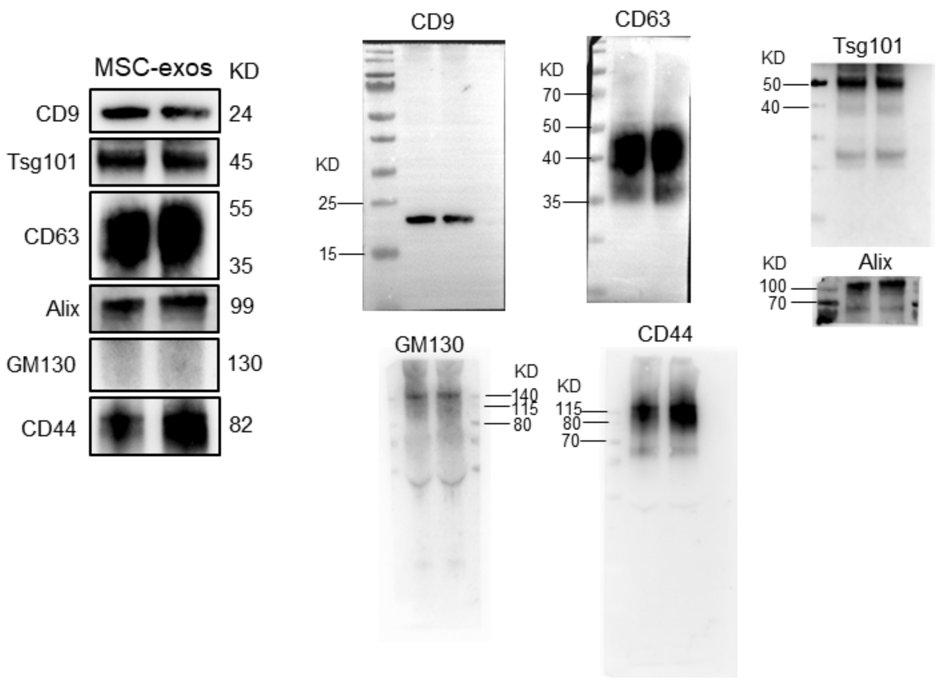

Unedited images for figure 2A

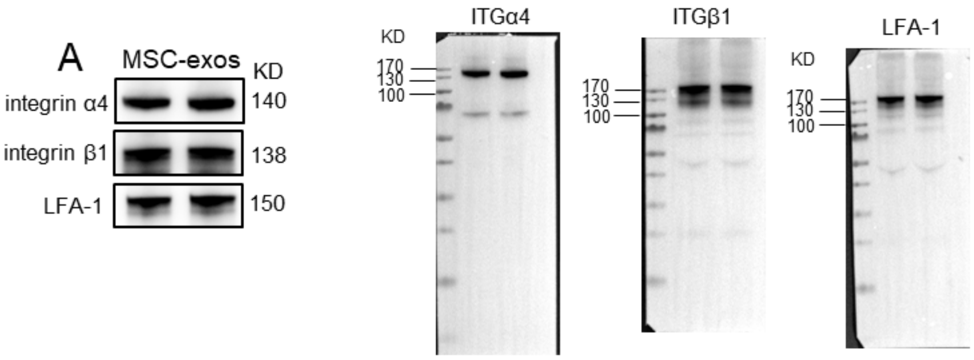

Unedited images for figure 2B

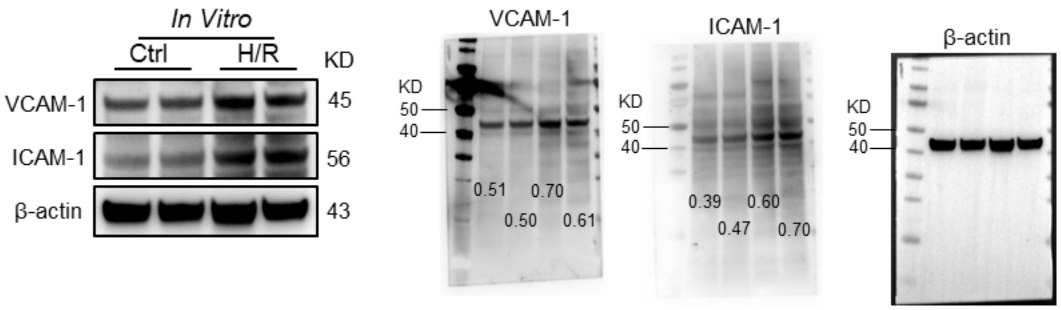

Unedited images for figure 2C

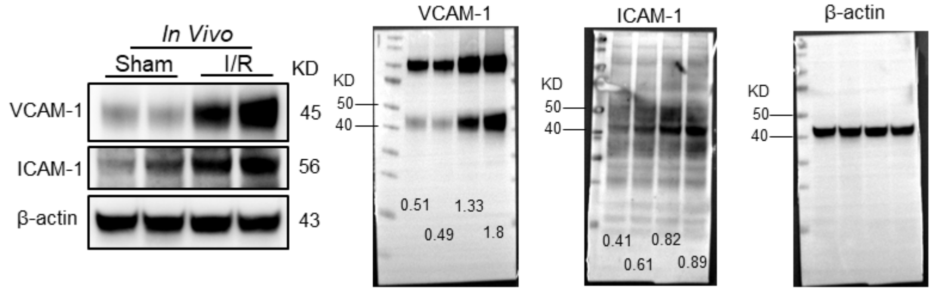

Unedited images for figure 4E

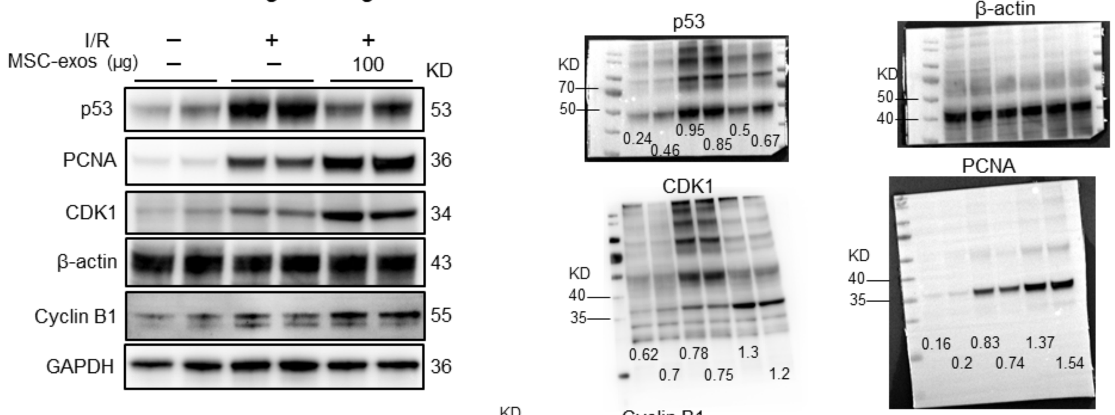

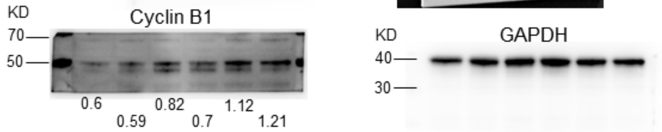

Unedited images for figure 4L

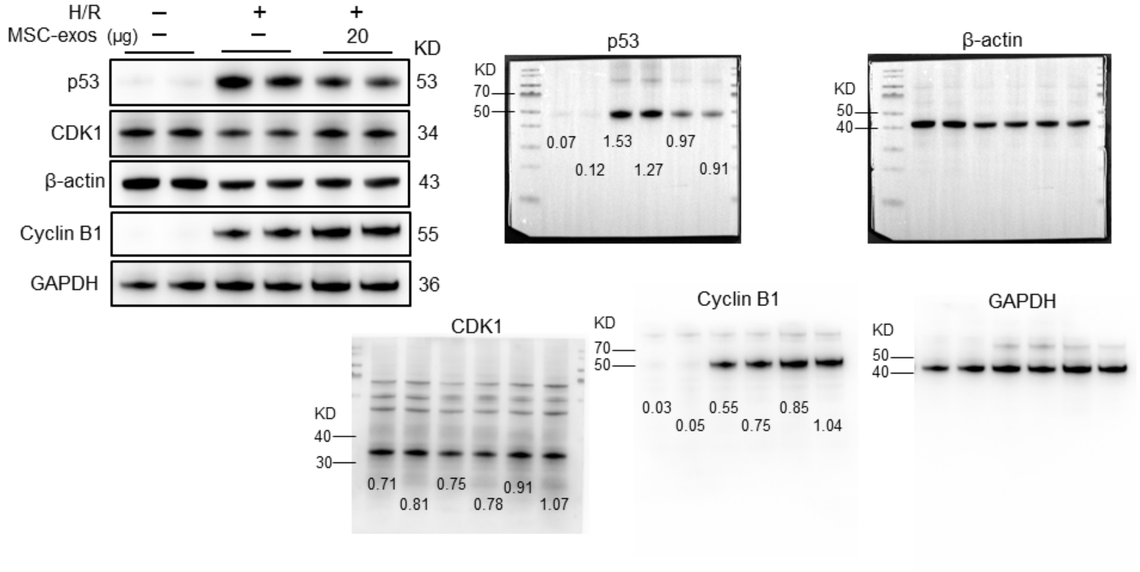

Unedited images for figure 5B

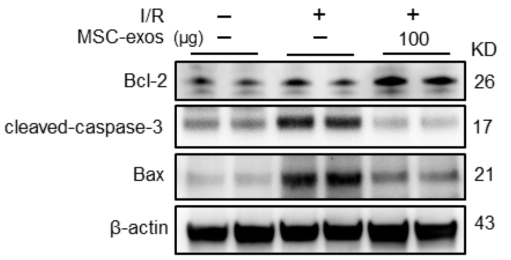

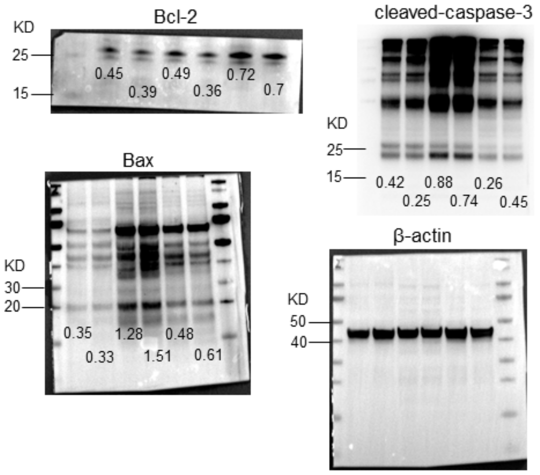

Unedited images for figure 5E

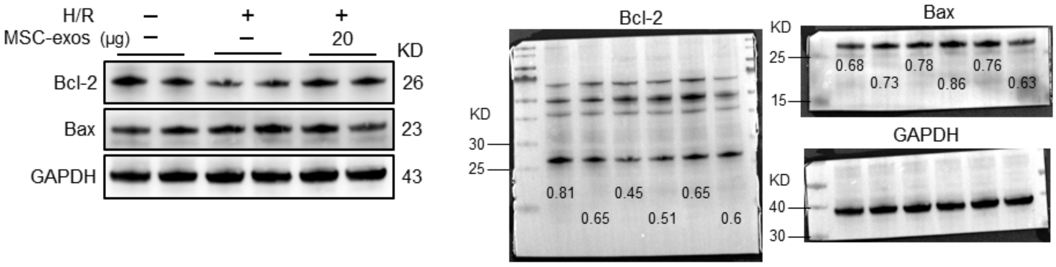

Unedited images for figure 7F

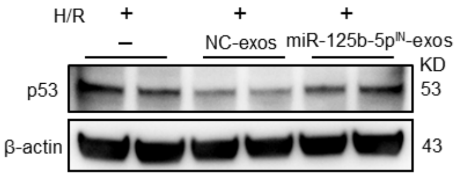

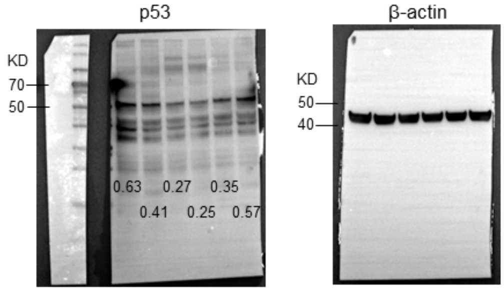

Unedited images for figure 7G

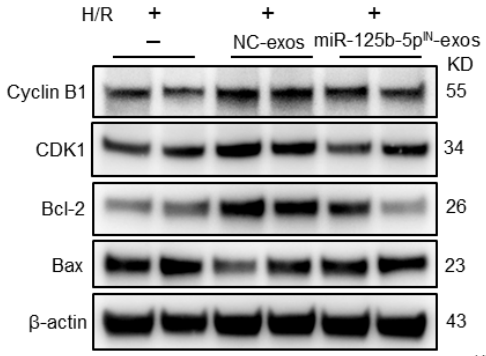

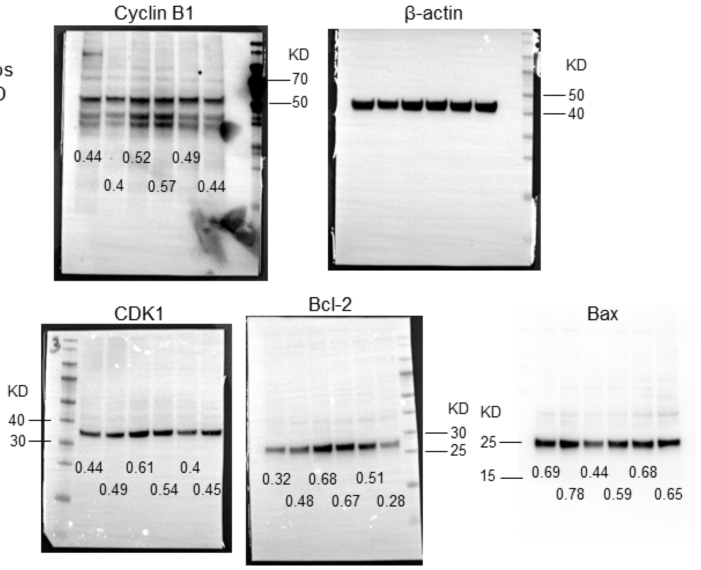

Unedited images for figure 8D

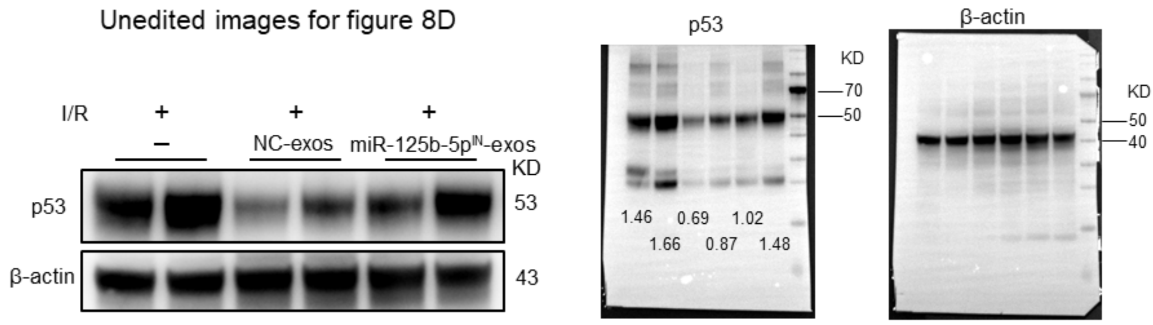

Unedited images for figure 8E

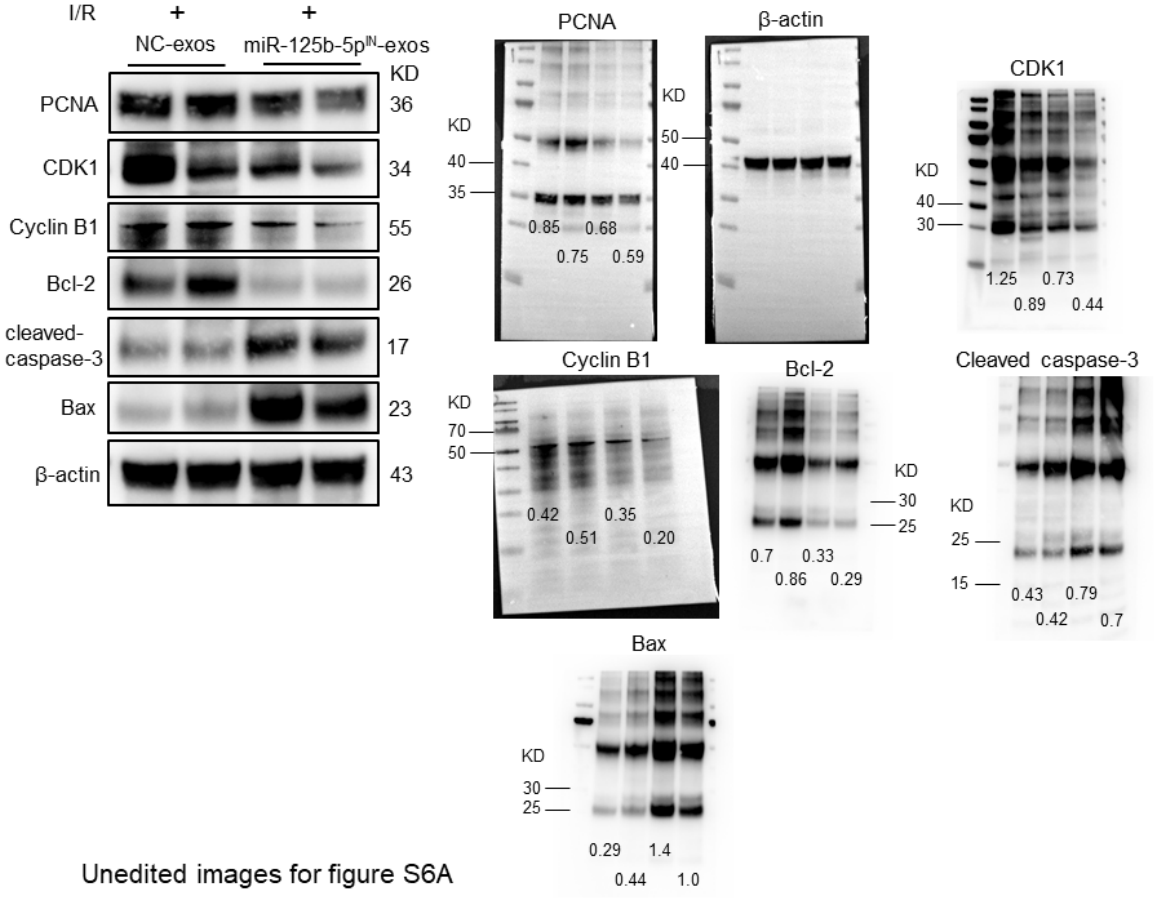

Unedited images for figure S6A

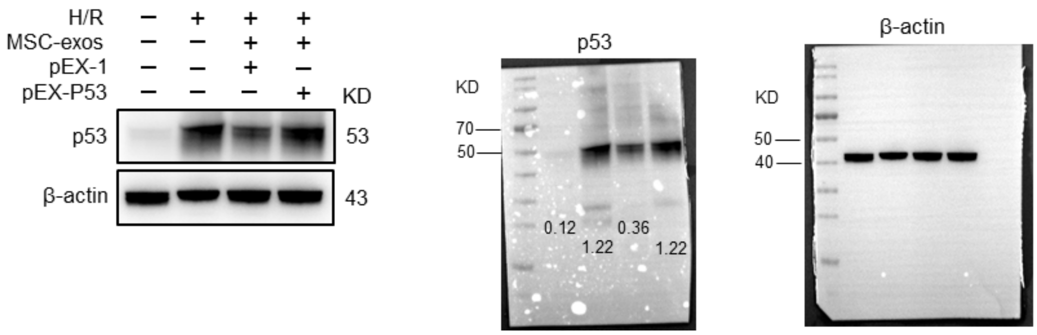

Figure S7. Unedited images of representative western blotting for figure 1-8 and S6. The data under

each band indicates that the gray value of the target band normalized to β-actin or GAPDH.

Supplementary table 1. Primers used in this study.

Gene	Forward	Reverse
β-actin-HOMO	CTACCTCATGAAGATCCTCACCGA	TTCTCCTTAATGTCACGCACGATT
p53-HOMO	AGCTTTGAGGTGCGTGTTTGTG	TCTCCATCCAGTGGTTTCTTCTTTG
β-actin-MUS	GGGAAATCGTGCGTGAC	AGGCTGGAAAAGAGCCT
MCP-1-MUS	TTGAGGTGGTTGTGGAAAAGG	GTGCTGACCCCAAGAAGGAAT
TNF-α-MUS	AGACAGAGGCAACCTGACCAC	GCACCACCATCAAGGACTCAA
IL-1β-MUS	GGTAAGTGGTTGCCCATCAGA	GTCGCTCAGGGTCACAAGAAA
IL-6-MUS	GTCACCAGCATCAGTCCCAAG	CCCACCAAGAACGATAGTCAA
